# Production of Food-Grade Monocalcium Phosphate from Meat-Bone Meal

**DOI:** 10.3390/ma18204653

**Published:** 2025-10-10

**Authors:** Zygmunt Kowalski, Agnieszka Wilkosz-Język, Agnieszka Makara

**Affiliations:** 1 Mineral and Energy Economy Research Institute, Polish Academy of Sciences, Wybickiego 7A, 31-261 Kraków, Poland; 2 Cracow University of Technology, Faculty of Chemical Engineering and Technology, Warszawska 24, 31-155 Kraków, Poland; a.wilkosz.jezyk@gmail.com (A.W.-J.); agnieszka.makara@pk.edu.pl (A.M.)

**Keywords:** meat-bone meal, monocalcium phosphate, feed-grade, hydroxyapatite, thermal processing

## Abstract

The study presents a developed process for producing monocalcium phosphate from hydroxyapatite ash, a by-product of meat-bone meal incineration. The process integrates technological and environmental synergies, enabling efficient recycling of both materials and energy. Waste hydroxyapatite ash, obtained as an intermediate by-product of the meat-bone meal process, is converted into high-quality monocalcium phosphate. Furthermore, waste heat from incineration is recovered, improving energy efficiency and reducing costs. Preliminary economic analysis indicates that the process is highly profitable, with an annual production capacity of 21,700 tons at a cost of $924 per ton, compared to a market price of $1400 per ton. The total production cost is estimated at $20,058,947, while total sales are projected to reach $30,380,000, yielding a profit of $10,321,053 (34% profit margin). The proposed method is consistent with the principles of the Circular Economy and Cleaner Production, promoting sustainability by reducing waste, lowering resource consumption, and enhancing energy efficiency. The developed technology is both environmentally friendly and economically viable, offering a promising pathway for efficient monocalcium phosphate production and a blueprint for industrial-scale implementation.

## 1. Introduction

Livestock farming, including the associated animal waste, is one of the largest sources of greenhouse gases (GHGs), accounting for approximately 14.5% of all anthropogenic GHG emissions [1,2]. The European Union (EU) is estimated to generate about 20 million tons of meat waste per year, consisting of 70% low-risk and 30% high-risk material [3]. A method for estimating meat waste generation in Europe was presented in [4]. The value chain of EU meat and meat waste produced in 2011 was as follows (million t/y): total meat waste 14.2, with primary production comprising 0.5, manufacturing 2.9, retail sales 1.7, household use 7.3, and food services 1.7.

Meat waste generated in the EU is mainly processed into meat-bone meal (MBM), the most widely applied industrial-scale valorization method [5,6]. The EU produces 4.5 million tons of MBM annually, primarily used as a biofuel [7,8]. MBM is also applied as a multi-component fertilizer, incorporated into the soil before sowing [9,10,11]. Depending on the application method, fertilization with MBM saves energy and can reduce GHG emissions to 1 ton of CO_2_ equivalents per 1 ton of MBM [12,13]. In 2021, Poland generated approximately 4.204 million t/y of meat waste, containing organic matter, water, and phosphorus compounds [14], which was processed into 0.967 million t/y of MBM, mostly used as a biofuel [15,16].

According to European legislation [17,18], meat waste is divided into three categories, reflecting different risk levels (high, medium, and low) to public and animal health, which determine the appropriate utilization or valorization methods. Proper meat waste management is closely linked to industrial symbiosis (IS), which promotes synergies in supply chain development and implementation to achieve economically efficient circular loops and increase resource yield through inter-firm cooperation in waste and energy flows. This approach supports the principles of the Circular Economy (CE), Cleaner Production (CP), and Sustainable Development (SD) [19,20]. At the microeconomic level, IS in industrial companies reduces primary resource consumption, fosters the reuse and recycling of waste, and helps prevent waste generation [21,22,23].

The CE model aims to maintain the highest possible quality and utility of products, components, and materials in biological and technical cycles. CP and SD are considered strategies within CE, applying CP methods to prevent environmental pollution throughout the product life cycle. CP technologies include source reduction, in-process recycling, on-/off-site recycling, and the substitution of natural resources with recycled materials [24,25,26].

Phosphorus recovered from animal waste represents a fraction of agricultural waste used in CE strategies in many countries [27,28]. Feed phosphates are inorganic compounds used in animal feeds [29]. They provide essential mineral nutrients for the development of strong bones, faster growth, and improved animal health [30]. Phosphorus (P) and calcium (Ca) are key nutrients for proper growth and bone mineralization [31]. In addition, calcium is crucial for eggshell formation, blood clotting, enzyme activation, and muscle contraction, while phosphorus plays an important role in cellular and membrane functions, fat and carbohydrate metabolism, and acid–base balance [32]. Although calcium and phosphorus are present in plant-based feedstuffs, inorganic feed phosphates (IFPs) are still required to meet the mineral needs of poultry [33].

Commercially used inorganic feed phosphates (IFPs) include monocalcium phosphate (MCP), dicalcium phosphate (DCP), defluorinated calcium phosphate (DFP), and tricalcium phosphate (TCP). Monodicalcium phosphate (MDCP) is a eutectic compound of MCP and DCP. It supplies phosphorus (P) and calcium (Ca), elements that are insufficiently provided in natural feed to meet animal nutritional requirements [34]. Compared with other feed phosphates, MDCP offers good phosphorus bioavailability, reduces phosphorus losses to the environment, and generates economic benefits [31]. The phosphorus availability in MCP is higher than in DCP, and in DCP it is higher than in TCP. Moreover, phosphorus availability in bone-derived DCP is greater than in rock-derived DCP. Bone-derived DCP and TCP are potentially cheaper and represent more sustainable sources of phosphorus compared with their rock-derived counterparts [35].

Feed phosphates are commonly produced using three technologies: wet, chemical, and thermal [30]. In the wet process, phosphoric acid reacts in a one- or two-stage process with calcium oxide or calcium carbonate. Depending on the phosphate type and intended use, the resulting product is dried after neutralization, crystallized, or granulated. The main products are MCP and DCP. The chemical process involves the reaction of phosphoric acid (in excess) with phosphate rock or calcium oxide, and most MCP and DCP are produced by this method. Defluorinated phosphate is obtained by reacting phosphate rock and sodium carbonate with phosphoric acid, followed by thermal defluorination [30,36]. If phosphoric acid reacts with lime and sulfuric acid, MCP and hydrated DCP are produced [32]. MCP, DCP, and MDCP differ in their phosphorus contents. To be classified as DCP, a product must contain ≥51% DCP. An IFP containing <80% but >51% MCP is classified as MDCP, while a product with >80% MCP is classified as MCP. Currently, one of the major challenges in IFP production is reducing production costs while maintaining essential process parameters and improving the physicochemical properties of the products for their intended applications.

Innovative developments in IFP production represent an important opportunity for implementing the Circular Economy (CE) strategy in the fertilizer industry [37,38]. The CE framework, supported by the European Commission (EC), prioritizes the sustainable use of natural resources [39]. The selection of an IFP manufacturing method must therefore be based on environmental, technological, and economic considerations [40].

The MBM combustion project developed for the Farmutil Company [41] involves the incineration of 30,000 t/y of meat-bone meal and the production of 7500 t/y of hydroxyapatite (HA)-rich ash. This high-quality HA is fluorine-free, and its conversion into phosphoric acid does not require complex defluorination, unlike phosphate rock. Cadmium concentrations are negligible, and the HA contains no radioactive compounds, which are present in trace amounts in all phosphate sources. The proposed CE concept builds on Cleaner Production (CP) principles, including in-process and off-process recycling of materials and energy obtained from MBM thermal treatment, as well as the substitution of natural phosphorites with recycled HA ash in phosphate production [42].

The research goal is to develop a technology for producing feed-grade MCP from ash obtained through MBM thermal processing. The scope of work includes: (i) characterization of the physicochemical properties of MBM and its thermal processing in laboratory and pilot-scale rotary kilns; (ii) determination of thermal processing parameters to obtain high-quality HA ash; (iii) development of MCP production technology based on HA ash, a product of MBM thermal utilization; (iv) identification of optimal process parameters; and (v) formulation of a conceptual process design for MCP production.

## 2. Materials and Methods

### 2.1. Analytical Methods

The moisture content of meat-bone meal (MBM) was determined using a WPS210S (Radwag, Radom, Poland) moisture analyzer at 105 °C, with a sampling interval of 5 s. Thermal analysis of MBM was carried out using an SDT 2960 Simultaneous DTA–DTG instrument (TA Instruments, Champaign, IL, USA) under an air atmosphere.

Ashes obtained from MBM and feed phosphates [43] were analyzed for total phosphate content, extracted using a 1:3 mixture of hydrochloric acid (HCl) and nitric acid (HNO_3_), as well as for soluble phosphates in 0.4% HCl and 2% citric acid. Extractions followed the standard method for phosphate determination in fertilizers [44]. Total and available phosphorus contents were quantified with a Marcel Media spectrophotometer (2 THETA, Český Těšín, Czech Republic), employing the differential photometric method based on the formation of a yellow phosphate–vanadate–molybdenum complex and absorbance measurement at 430–450 nm.

Calcium content in MBM samples was measured by atomic absorption spectroscopy (AAS) using a PerkinElmer Analyst 300 instrument (Perkin Elmer, Springfield, IL, USA). Measurements were performed in an air–acetylene flame at 422.6 nm, with calibration against standard solutions. Calcium content in hydroxyapatite ash samples was determined following analytical methods for phosphates [44]. The procedure involved dissolution in nitric acid, precipitation of phosphates as bismuth(III) phosphate(V) (BiPO_4_), and complexometric titration of calcium with ethylenediaminetetraacetic acid (EDTA) in the presence of a mixed indicator (fluorexone and thymolphthalein).

The phase composition of raw MBM, ashes, and resulting phosphates was identified using X-ray diffraction (XRD) with a Philips X’Pert diffractometer equipped with a PW 1752/00 graphite monochromator (Philips Analytical, Nederweert, The Netherlands). Scanning electron microscopy (SEM) coupled with energy-dispersive X-ray spectroscopy (EDS) was used to assess the effect of MBM processing temperature on the quality of hydroxyapatite ash. SEM analysis was conducted with a Hitachi S-4700 electron microscope equipped (Hitachi High-Technologies Corporation, Tokyo, Japan) with a microanalysis system.

The contents of heavy metals and other elements were determined by inductively coupled plasma optical emission spectroscopy (ICP-OES) using a Philips Scientific PU 7000 spectrometer (Philips, Amsterdam, The Netherlands) equipped with an ultrasonic nebulizer Cetac At-5000 (Teledyne CETAC Technologies, Omaha, NE, USA).

The mean values with standard deviations (SD) were calculated from three replicates, and statistical significance was assessed using ANOVA, where *p* < 0.05. In the case of elemental analysis by ICP for meat-bone meal (MBM) and hydroxyapatite ash (HA) after calcination, measurement uncertainties were estimated [45].

### 2.2. Utilization of Meat Waste to Produce MBM

#### 2.2.1. MBM Properties

One ton of meat-bone meal is produced from approximately four tons of meat waste using the hydrothermal method. Due to the heterogeneity of meat waste, the characteristics of the resulting MBM can vary considerably [15,41], and its composition depends on the type of raw material used. The waste products utilized in MBM production include hides, skins, blood, rumen contents, bones, horns, hooves, urinary bladder, gall bladder, uterus, rectum, udder, fetuses, snout, ears, penis, meat trimmings, hide and skin trimmings, condemned meat and carcasses, esophagus, hair, and poultry offals (such as feathers and heads).

The extent to which all by-products can be used depends on several factors. According to [46], effective utilization of animal by-products requires:

- A commercial process for converting the by-product into a usable commodity;
- An actual or potential market for the commodity;
- Sufficient volumes of economically priced material in one location for processing;
- Appropriate facilities for storing the perishable by-products before processing and the finished products after processing;
- A critical mass of trained technical operators.

Farmutil processes over 600,000 t of meat waste annually in MBM units, which rank among the most modern in the EU due to advanced technology and equipment solutions. This allows utilization of over 60% of meat waste produced in Poland, ensuring that the above conditions are met [47].

In practice, most raw materials in the MBM production process are mixed, which averages out their composition and significantly reduces variability. This applies to approximately 90% of the material processed into meat-bone meal (MBM) and bone meal (MB). Only blood meal (BM) is usually processed separately. According to the relevant standard, MBM and MB typically contain about 9% P, and their quality is strictly controlled to comply with the Animal Feeding Stuffs standard [48].

MBM is commonly used in low-cost pet food formulations due to its high protein content. However, its nutritional properties can fluctuate significantly, and supplementary processing is often required [49]. The methods for manufacturing meat meals are well documented in the literature [50,51].

After pretreatment (removal of metals, shredding, and mixing), the meat waste is sterilized at 133 °C and 0.3 MPa for 30 min, then dried, followed by fat separation through filtration. The solid fraction obtained after filtration is subsequently ground and sieved. This process complies with EU regulations [51]. Meat waste is classified into three risk categories [5,18], which determine the corresponding categories of the MBM produced.

Possible elimination or valorization methods for MBM include: combustion or co-combustion (categories 1–3), landfill disposal (categories 2–3), biogas production (categories 1–3, after sterilization), and use as biofuels (categories 1–3), organic fertilizers (categories 2–3), or animal feed (category 3). According to [10,48,50], animal-origin meals (Table 1) can be classified by protein content (40–89%) into meat meal (MM), MBM, bone meal (MB), blood meal (BM), skin meal (SM), and liquex meal (LM). The antioxidant content should range from 100 to 400 mg/kg.

#### 2.2.2. MBM Incineration Process Description

**Figure 1:**
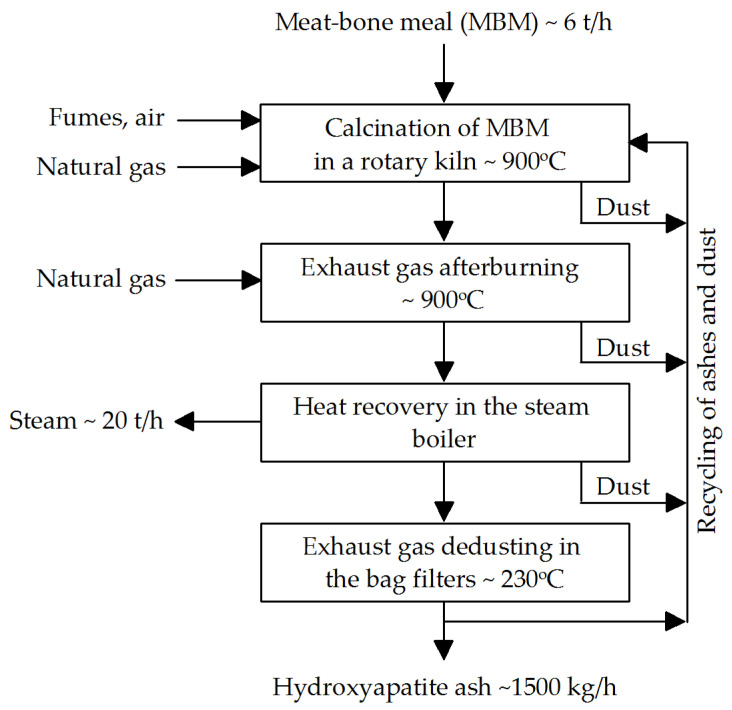
Flow sheet of the meat-bone meal (MBM) thermal processing.

The process parameters of MBM combustion in a rotary kiln (Figure 1) are as follows [41]:

- Calcination time: 30–60 min for a feed rate of approximately 50 kg/m^2^/h.
- Maximum feedstock temperature: 950 °C.
- Co-current rotary kiln operation with adjustable rotation speed of 1–2 rpm.
- Gas flow velocity in the rotary kiln: up to 4 m/s.
- Oxygen concentration in exhaust gases after the rotary kiln: ~11%.
- Mass ratio of recycled HA to MBM in the feed charge: 1:1; recycled HA can be introduced via the kiln dosing screw.
- In the afterburner chamber, exhaust gases are combusted within 3 s at 850–900 °C.
- Steam produced in the steam boiler: pressure 6 bar.
- Exhaust gas flow rate during dedusting in bag filters: ~2 m/s at 200–250 °C.
- Specific consumption per 1000 kg of HA produced: 4000 kg MBM, 80 kWh electricity, 0.1 m^3^ process water, and 90 m^3^ natural gas.

In study [15], MBM samples were calcined in a chamber kiln. The combustion heat of MBM was 18.5 MJ/kg. The weight loss during calcination was 70.0%. The phosphorus content ranged from 14.5% to 15.0%, while the calcium content ranged from 33.6% to 33.8%. Phase composition analysis showed that the main crystalline phase of MBM was hydroxyapatite (Ca_5_(PO_4_)_3_OH), with trace impurities including SiO_2_, Ca_3_(PO_4_)_2_, and CaCO_3_. The phosphorus content in HA was comparable to that in natural phosphorites (13.2–17.2% P) [16]. Water removal is a crucial step in rendering, with the resulting aqueous stream accounting for approximately 65% of the waste mass [51]. The meat waste used for MBM production typically consists of ground pork bones measuring 1–3 cm, with a water content of 35.0–45.0%. The dry mass composition includes (in % of dry matter): organic matter 34.0–39.0, fat 14.0–16.0, protein 18.0–23.0, P 10.0–14.0, and Ca 28.0–30.0. The bulk density of MBM is 0.85 kg/dm^3^. The concentrations of Cd, Hg, As, Cr, Pb, and Cu are all below 0.1 ppm, confirming the high quality of HA and its very low heavy metal content [15]. All types of MBM can be combusted in a rotary kiln.

Research [16] demonstrated that by reducing the MBM calcination temperature to below 950 °C, recycling hydroxyapatite (HA), and mixing it with MBM at a 1:1 ratio, the carbon content in the resulting HA ash can be reduced to <0.2%, while P_2_O_5_ content can increase up to 39%. This high-grade hydroxyapatite product could serve as a substitute for natural phosphorites. Furthermore, MBM incineration also generates bioenergy, illustrating the implementation of a highly profitable Circular Economy (CE) approach.

## 3. Results

### 3.1. Properties of MBM and Ashes Obtained from Thermal Processing of MBM: Laboratory Test Results

**Figure 2:**
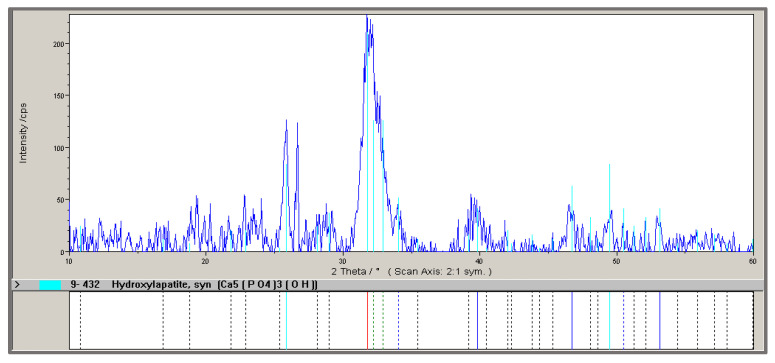
X-ray diffraction pattern of a meat-bone meal sample.

MBM samples were analyzed for moisture, phosphorus (P) and calcium (Ca) contents, heat of combustion, and phase composition (Figure 2). The average composition of MBM is as follows (in %): maximum moisture 10.0, P 5.5–6.0, and Ca 7.0–8.0. The average heat of combustion of MBM is 18.5 MJ/kg, and its bulk density is 0.6 t/m^3^. Combustion of MBM produces hydroxyapatite (HA) with a yield of 25–30%. Phase composition analysis (Figure 2) confirmed that the primary crystalline phase of MBM is hydroxyapatite.

**Figure 3:**
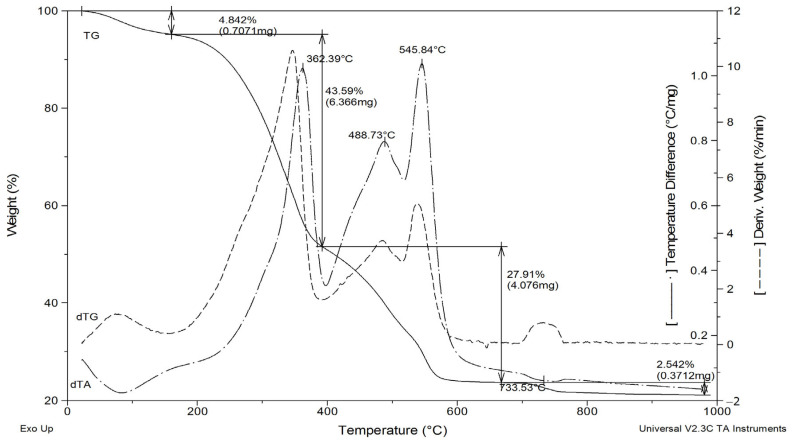
Thermal analysis (TGA-DTA) of the meat-bone meal sample.

Thermal analysis (TGA–DTA) (Figure 3) shows that MBM undergoes thermal decomposition in three distinct stages. The process involves ignition and decomposition of the organic components, as evidenced by a pronounced exothermic effect. Three characteristic regions can be distinguished in the obtained curves. Dehydration occurs up to 148 °C. The weight loss observed between 148 °C and 225 °C is associated with the evaporation of low-molecular-weight compounds and initial decomposition reactions. The highest rate of weight loss occurs between 200 °C and 400 °C, corresponding to the degradation of organic substances. The peaks observed between 360 °C and 500 °C are likely associated with the thermal decomposition of bone components. MBM undergoes thermal degradation up to approximately 550 °C, corresponding to the combustion of organic materials, as indicated by a pronounced exothermic effect. The weight loss attributed to the combustion of the organic phase of MBM is about 76%. The average moisture, phosphorus (P), and calcium (Ca) contents in MBM samples were 2.43%, 5.8%, and 7.7%, respectively.

The peaks observed at around 750 °C correspond to the endothermic decomposition of CaCO_3_ present in the ash. MBM incineration converts 25–30% of the initial MBM mass into hydroxyapatite (HA) ash. The primary crystalline phase of the ash is hydroxyapatite, which is homogeneous in terms of composition and chemical properties. The high purity of HA indicates an absence of significant heavy metals.

#### 3.1.1. Properties of Hydroxyapatite Ashes Obtained from MBM in a Laboratory Chamber Kiln

MBM samples were calcined in a chamber kiln for 3 h at 600 °C and 950 °C. This temperature range was selected based on thermal analyses of MBM (see Section 3.1) and previous research [16], which indicated that the maximum calcination temperature for MBM should remain below 950 °C. The phosphorus (P) and calcium (Ca) contents, as well as the phase composition of the resulting ashes, are presented in Table 2.

The P content of the product is typical of natural phosphorites, ranging from 13.2% to 17.2% [42]. The particle size distribution of the ash is as follows: >0.25 mm—65%; 0.16–0.25 mm—15%; 0.10–0.16 mm—7%; <0.10 mm—13%. The bulk density is 0.7 t/m^3^. X-ray diffraction analysis indicated that the primary phase of the obtained ash is hydroxyapatite (Ca_10_(PO_4_)_6_(OH)_2_), while minor phases include silica (SiO_2_), tricalcium phosphate (Ca_3_(PO_4_)_2_), and calcite (CaCO_3_).

Table 2 presents the characteristics of hydroxyapatite ash obtained from meat-bone meal (MBM) after calcination at two different temperatures: 600 °C and 950 °C. An increase in the calcination temperature leads to a higher weight loss, which is 70% at 600 °C and 77% at 950 °C, indicating more intense decarboxylation processes and gas release at higher temperatures.

The phosphorus content in the ash is 14.5 ± 0.5% at 600 °C and 15.0 ± 0.4% at 950 °C, showing a slight change in the phosphorus content due to calcination at these temperature ranges. However, the increase in calcination temperature results in a higher calcium content, which is 33.8 ± 0.4% at 600 °C and 36.6 ± 0.6% at 950 °C. This phenomenon is associated with the decarboxylation of calcium carbonate and its transformation into more stable calcium forms.

The phase composition analysis reveals the presence of hydroxyapatite (Ca_10_(PO_4_)_6_(OH)_2_) as the dominant phase at both 600 °C and 950 °C. In the samples calcined at 600 °C, phases such as SiO_2_, calcium phosphate (Ca_3_(PO_4_)_2_), and calcium carbonate (CaCO_3_) are also present, whereas in the samples calcined at 950 °C, calcium carbonate completely disappears, suggesting its decarboxylation. In this case, SiO_2_ and calcium phosphate (Ca_3_(PO_4_)_2_) are also present, indicating structural stability at the higher temperature.

**Figure 4:**
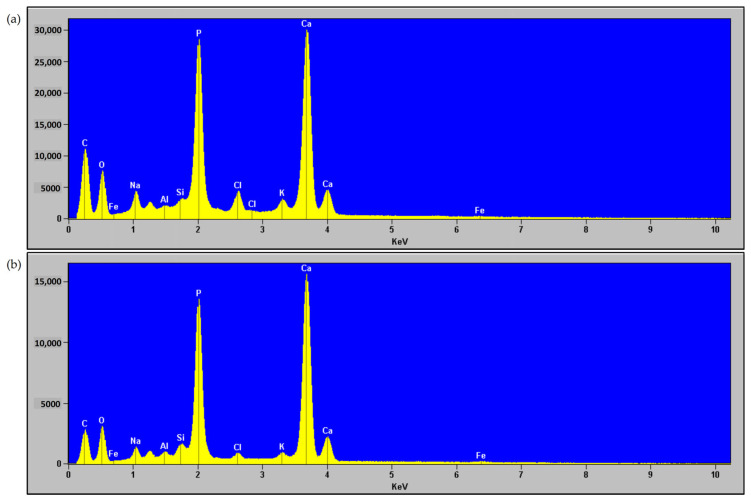
EDS spectra of hydroxyapatite ash obtained by incinerating meat-bone meal for 3 h at temperatures: (**a**) 600 °C, (**b**) 950 °C.

**Figure 5:**
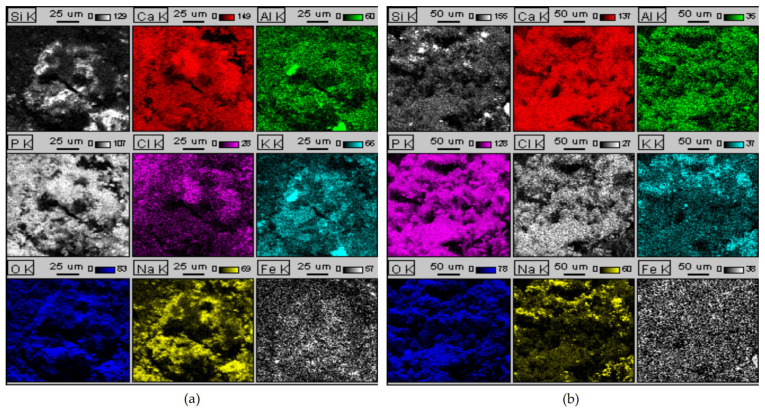
Elemental distribution maps of hydroxyapatite ash obtained by calcining meat-bone meal for 3 h at [°C]: (**a**) 600, (**b**) 950.

**Figure 6:**
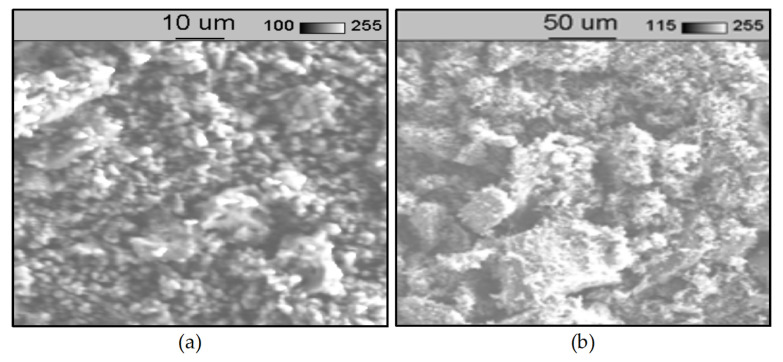
SEM images of hydroxyapatite ashes obtained by calcining meat-bone meal for 3 h at temperatures [°C]: (**a**) 600 (magnification 1800×), (**b**) 950 (magnification 500×).

Figure 4 and Figure 5 present the elemental distribution maps (EDS) and spectra of hydroxyapatite (HA) produced by incinerating meat-bone meal for 3 h at 600 °C (lowest) and 950 °C (highest), respectively. Scanning electron microscopy (SEM) images of the HA are shown in Figure 6.

The EDS spectra of both hydroxyapatite (HA) ashes (Figure 4) reveal the presence of the following elements: carbon, oxygen, iron, sodium, aluminum, silicon, phosphorus, chlorine, potassium, and calcium. The two most prominent peaks correspond to phosphorus (P) and calcium (Ca), the primary constituents of hydroxyapatite, as also confirmed by X-ray analysis. Other elements, including silicon, sodium, aluminum, potassium, iron, and chlorine, should be considered as contaminants in the analyzed samples. EDS spectra with the same elemental composition were obtained for HA ashes produced at 600 °C and 950 °C.

Elemental distribution maps indicate that Ca and P are the main components in both HA ashes, uniformly distributed throughout the samples, confirming hydroxyapatite as the dominant mineral phase. The presence of additional elements such as Si, Na, K, Fe, Cl, and Al should be interpreted as impurities originating from the raw material. In the sample calcined at 600 °C, the distribution of trace elements is more heterogeneous, possibly resulting from incomplete mineralization of organic residues. In contrast, the sample obtained at 950 °C exhibits greater homogeneity and a more uniform elemental distribution, indicating a higher degree of purity of the HA ash produced at this temperature.

The surface of the ash sample calcined at 600 °C (Figure 6a) is fine-grained and composed of numerous small, irregularly shaped particles. Residual traces of incomplete mineralization are visible, suggesting the presence of residual organic structures or uneven crystallization of the hydroxyapatite phase. In contrast, the sample calcined at 950 °C (Figure 6b) exhibits a more consolidated structure, with larger, denser agglomerates and well-defined grain boundaries. These observations indicate that the higher calcination temperature promoted recrystallization, resulting in a more homogeneous mineral phase, improved thermal stability, and a higher degree of purity in the obtained ash.

Table 3 presents the concentrations of heavy metals and other elements in hydroxyapatite (HA) obtained by incinerating MBM at 750 °C for 3 h, compared with their concentrations in the original MBM. All determinations were performed using inductively coupled plasma (ICP) spectroscopy.

The analysis of meat-bone meal (Table 3) before and after calcination at 750 °C reveals significant changes in elemental composition. The Cd content in meat-bone meal (MBM) is below 0.002 mg/kg, and the As content is below 0.01 mg/kg. In HA ash, these values increase mainly due to the loss of organic matter and water during the thermal processing of MBM, reaching 0.014 mg/kg for Cd and 0.84 mg/kg for As. Importantly, these concentrations remain well below the maximum permissible levels in feed phosphates specified by the standard [48], which sets the limit for both As and Cd at 10 mg/kg. Specifically, the As concentration is about 12 times lower, and the Cd concentration about 724 times lower than the regulatory thresholds. For comparison, Cd levels in natural phosphorites typically range from 7 to 52 mg/kg [42]. Mercury (Hg), however, is largely volatilized during combustion, while lead (Pb) content remains essentially unchanged at 1.3 mg/kg. Copper (Cu), zinc (Zn), and silicon (Si) show notable enrichment, indicating their stability within the mineral phase. Phosphorus (P) content increases markedly from 4.18% to 17.9%, reflecting the enrichment of the hydroxyapatite phase in the ash. In contrast, calcium (Ca), magnesium (Mg), potassium (K), and sodium (Na) exhibit substantial decreases, associated with volatilization during combustion. Nitrogen (N) is almost entirely lost, decreasing from 8.10% to 0.16%, which is typical for high-temperature calcination processes. Chloride (Cl^−^) content also decreases significantly, whereas fluoride (F^−^) is partially retained and becomes concentrated in the HA.

Overall, incineration of meat-bone meal at 750 °C results in the enrichment of stable elements and the formation of a more homogeneous mineral phase characteristic of hydroxyapatite ash, accompanied by the loss of volatile components.

#### 3.1.2. MBM Incineration with In-Process Recycling of HA: Laboratory Chamber Kiln Tests

The parameters for thermal treatment of MBM [16,41] indicated that the recommended conditions for MBM incineration in a rotary kiln are a calcination time of 30–60 min and a material temperature of ≤950 °C. Initial combustion tests were performed using mixtures of MBM and recycled hydroxyapatite (HA) obtained by incinerating MBM at 950 °C for 3 h. These tests were conducted in a chamber kiln for 3 h at 950 °C. The phosphorus (P) and calcium (Ca) contents of the resulting ashes were determined (Table 4), and their phase composition was analyzed using X-ray diffraction (XRD). XRD analysis confirmed that the calcined HA ash contained the following phases: Ca_5_(PO_4_)_3_OH, Ca_3_(PO_4_)_2_, SiO_2_, and Fe_2_O_3_.

Subsequent tests on MBM incineration with in-process recycling of hydroxyapatite (HA) were carried out, investigating variables such as the MBM-to-HA mass ratio, temperature, and calcination time. The experimental conditions and the resulting HA ash composition are presented in Table 5.

**Figure 7:**
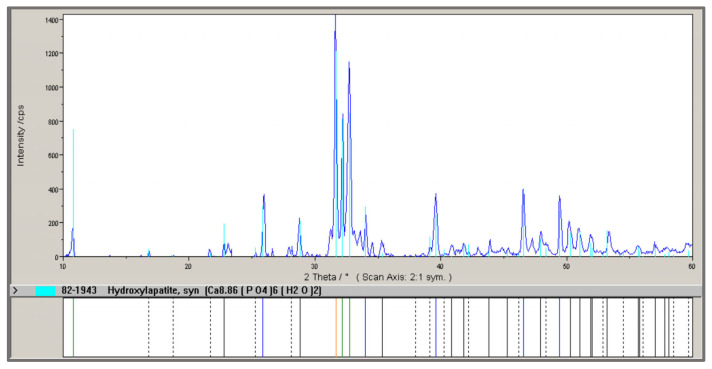
X-ray diagram of hydroxyapatite ash obtained after calcining meat-bone meal (MBM) and hydroxyapatite ash (HA), with an MBM:HA mass ratio of 1:5.5, at 950 °C, for 105 min (sample 12).

The X-ray diffraction (XRD) pattern of the HA obtained in sample 12 is shown in Figure 7. Hydroxyapatite is the primary crystalline phase present in all samples. The intensity of the characteristic hydroxyapatite peaks is consistent across all tested samples, indicating a similar degree of crystallization regardless of the calcination temperature, duration, or the MBM-to-recycled HA mass ratio.

### 3.2. Quarter-Scale MBM Incineration Test Results

**Figure 8:**
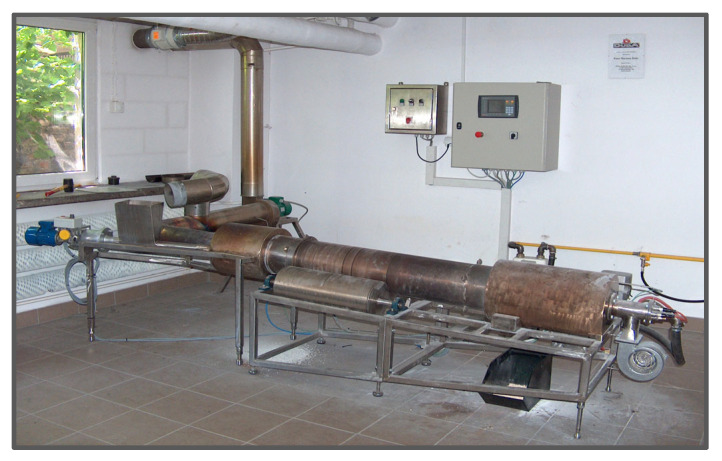
Laboratory rotary kiln.

**Figure 9:**
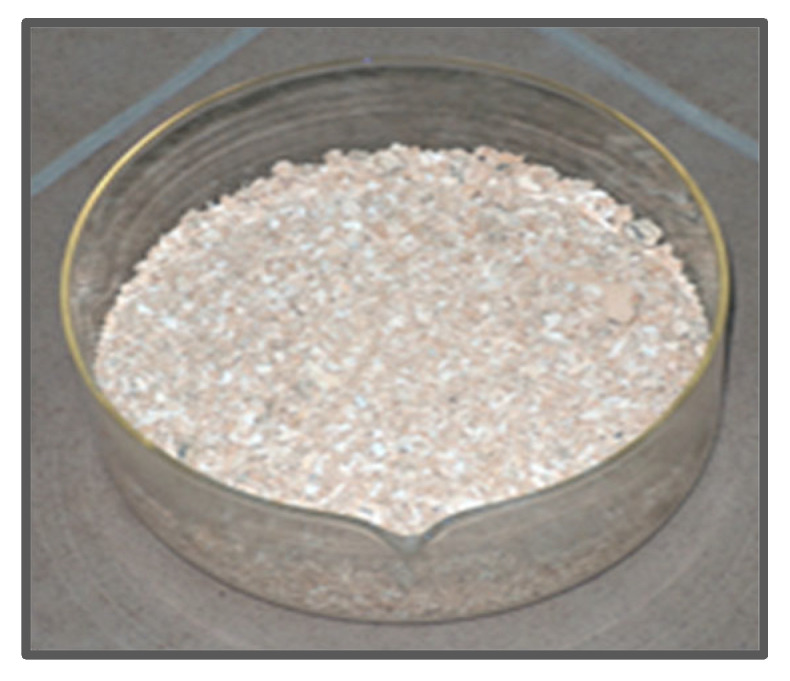
Hydroxyapatite ash from calcining meat-bone meal in a laboratory rotary kiln at 800 °C.

MBM combustion tests were carried out in a laboratory-scale co-current flow rotary kiln (Figure 8). The resulting hydroxyapatite (HA) ash formed small agglomerates a few millimeters in size, exhibiting two distinct colors: beige and white (Figure 9). The kiln is equipped with a ribbon feeder for dosing the charge. The calcination process was conducted at approximately 800 °C, measured at the kiln wall where the MBM was fed, while the inlet temperature reached about 1050 °C. At the measurement point located before the exhaust fan and after the cold air inlet (used to extract odors from the feeder hopper), the temperature ranged from 270 to 320 °C. The furnace rotation speed was 1.5–2 rpm, and the feeder speed ranged from 25 to 35 rpm. The material remained in the kiln for 20–30 min. The kiln dimensions were 1200 mm in length and 150 mm in diameter.

Incineration tests were carried out in a rotary kiln using a mixture of MBM and recycled hydroxyapatite (HA) at 600 °C and 800 °C. The phosphorus (P) and calcium (Ca) contents of the resulting HA products were determined, and the phase composition of the ash was analyzed. The results are presented in Table 6.

The primary crystalline phase of all obtained ashes is hydroxyapatite (HA). The peak intensities corresponding to the hydroxyapatite phase are similar across all tested samples, indicating a comparable degree of HA crystallization, regardless of calcination temperature, duration, or the MBM-to-recycled HA mass ratio.

**Figure 10:**
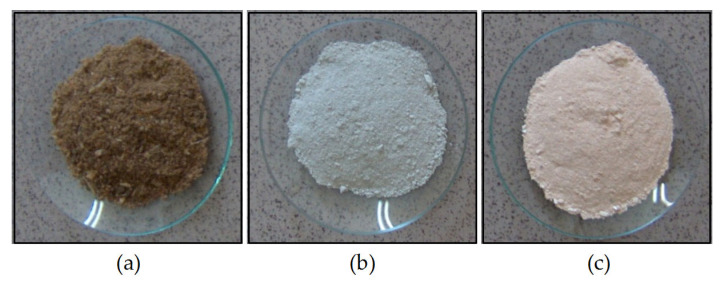
Images of the samples: (**a**) Meat-bone meal, (**b**) Hydroxyapatite ashes obtained after calcining MBM at 600 °C, (**c**) Hydroxyapatite ashes obtained after calcining MBM at 800 °C.

Incorporating recycled HA into MBM significantly facilitates feeding the material into the kiln. The MBM-HA mixture is more free-flowing than MBM alone, preventing clumping and ensuring smooth operation of the ribbon feeder. At 800 °C, uniform beige HA was obtained, whereas at 600 °C, gray HA was produced. Images of the MBM samples and the resulting ashes at 600 °C and 800 °C are shown in Figure 10.

The color change in hydroxyapatite to yellow or orange hues is mainly due to doping with iron, which leads to changes in the material’s structure and optical properties. Iron in the form of Fe^3+^ or Fe^2+^ replaces calcium in the crystal lattice, causing a change in light absorption and imparting a new color [52]. Additionally, calcination processes can result in the formation of new phases (e.g., iron oxides), and the presence of carbon can influence the color of the material through light absorption. The presence of other metal impurities in the HA structure may also affect the color through mechanisms related to the formation of new phases or interactions with iron ions [16].

The phosphorus (P) content in the obtained hydroxyapatite (HA) is comparable to that of typical phosphorites, ranging from 13.2% to 17.2%. The grain size distribution of the product is as follows: >0.25 mm—65%, 0.16–0.25 mm—15%, 0.10–0.16 mm—7%, and <0.10 mm—13%. The bulk density is 0.7 t/m^3^. All types of MBM can be incinerated in a rotary kiln. On average, MBM contains ≤10.0% moisture, 5.5–6% P, and 7–8% Ca. The average calorific value of MBM is 18.5 GJ/t, and its bulk density is 0.6 t/m^3^. X-ray analysis indicated that the primary constituent of MBM is hydroxyapatite.

### 3.3. Characteristics of Inorganic Feed Phosphates (IFP)

Before evaluating the quality of inorganic feed phosphates (IFPs), it is important to understand their naming conventions. Typically, monocalcium phosphate (MCP) contains approximately 22.7% phosphorus (P), mono-dicalcium phosphate (MDCP) contains about 21% P, and dicalcium phosphate (DCP) contains around 18% P. Calcium (Ca) content in MCP and MDCP generally ranges from 15% to 18%, whereas DCP contains approximately 24% Ca. These values may vary slightly depending on the quality of the acid used and the production method. The P content alone is not sufficient to determine the phosphate type; the Ca:P ratio is a useful tool for categorizing and quickly identifying the IFP composition in feed material catalogs [53].

For IFP assessment [54,55], two main categories of parameters should be considered: nutritional parameters, which evaluate feed phosphate quality from a nutritional perspective, and technological parameters, which indicate the product’s stability and potential chemical reactivity risks during storage or mixing.

Granulometric analysis determines the distribution of particle sizes in a sample. The uniformity of granulometry can significantly influence the homogeneity of the final product. Particle size also affects flowability, caking risk, and chemical reactivity. Smaller particles tend to present higher risks, such as increased caking or reactivity. Feed phosphate products can have different granulometric types—granular, micro-granular, or powder—each corresponding to a specific particle size range: granular, 0.5–2 mm; micro-granular, 0.2–1.8 mm; powder, 0–0.25 mm.

Flowability refers to a powder’s ability to flow freely, smoothly, and consistently as individual particles. Poor flowability may lead to clumping, which can damage machinery. The ideal flowability value is 100, and it can be influenced by factors such as water content, particle size, humidity, and temperature.

Several methods can be used to evaluate powder flowability:

- Angle of repose: measured after the powder is poured into a heap. Smaller angles indicate better flowability.
- Tapping tests: used to evaluate the bulk density of the powder. Powders that compact more easily generally exhibit better flowability.
- Rheological tests: assess the resistance of the powder to flow under various conditions of stress and deformation.
- Shear cell measurements: apply shear stress to the powder, measuring the force required to induce movement, providing a precise quantitative assessment of flowability.

Friability refers to the degradation of granules due to physical actions such as mechanical shocks or friction. Excessive friability can lead to dust formation, negatively affecting manufacturing, packaging, storage, and transportation. Ideally, friability should be less than 1%. For example, MCP typically exhibits a friability of around 0.5%.

The pH of a phosphate should be considered in relation to the intended use of the end product, as it affects enzyme function and nutrient solubility in the stomach. Ruminants have a neutral to slightly basic stomach pH of 6–7, while monogastric animals have an acidic stomach pH of 2–3 [50,51]. Therefore, feed for pigs and poultry preferably contains low-pH phosphates to protect the intestines from pathogens and enhance nutrient solubility. Acidifying agents may also be applied to maintain optimal stomach pH, supporting proper proteolytic enzyme activity and controlling intestinal microflora. However, acidic phosphates can be corrosive, increasing the risk of accelerated machinery wear—particularly when switching from basic to acidic phosphates, which is important for mineral feed manufacturers. Each IFP has a specific pH or a defined pH range. For instance, MCP with 22.7% P has a pH of 3, whereas DCP with 18.0% P has a pH of 9.53.

Phosphorus water solubility (Pws) indicates the proportion of phosphorus soluble in water. MCP typically has a Pws above 75%, MDCP above 65%, and DCP (mainly composed of DCP molecules) below 10%. A higher MCP content increases solubility and digestibility, particularly for pigs, poultry, and aquatic species.

Reactivity is measured by the temperature increase within five minutes when phosphate reacts with water. Highly reactive products generate a higher temperature rise (exothermic reaction), indicating potential risks of chemical interactions with other feed components, such as magnesium oxide. Low to medium reactivity is generally preferred for mineral premixes and lick blocks, whereas high reactivity in typical feed or loose licks may not pose significant problems.

Free acidity reflects the content of H_3_PO_4_, while CO_2_ content indicates the quantity of CaCO_3_ in the phosphate. High free acidity suggests incomplete reaction between phosphorus and calcium. This is particularly critical for hygroscopic phosphates like MCP, where high free acidity can increase the risk of caking. Both free acidity and CO_2_ content are valuable for monitoring technological processes, as excessive free acidity can exacerbate caking.

Humidity at 100 °C represents the free water content of the product, which should not exceed 2%. High humidity increases the risk of chemical reactions, caking (reducing flowability), and poor storage stability. Loss on ignition (LOI) at 250 °C or 550 °C measures water bound to phosphate molecules. Calculating the difference between LOI and humidity at 100 °C allows determination of whether DCP is anhydrous or dihydrated. A value below 10% indicates anhydrous DCP, whereas 10% or higher indicates dihydrated DCP, which will lose additional water molecules between 100 °C and 250 °C. Humidity also affects heavy metal measurements in IFPs and should be standardized at 12% for such calculations.

#### Quality Classification of Inorganic Feed Phosphates (IFP)

Inorganic feed phosphates (IFPs) are the preferred source of phosphorus (P) in animal feed due to their high P quality. In contrast, P from cereals is low, highly variable, and largely indigestible by animals. Mineral P from IFPs is less affected by external factors. Most IFPs are extracted from phosphate rock and processed to improve P digestibility for animals. These IFPs are consistent in composition, have low levels of impurities, and are considered the best available source of P for animal nutrition. Accurate calculation of P levels to meet animals’ nutritional requirements is crucial from both nutritional and environmental perspectives [56].

The quality of feed phosphates can vary significantly in terms of chemical composition, which is strongly correlated with P digestibility and purity, i.e., the content of undesirable elements. In the EU, maximum permissible levels for Pb, Cd, Hg, As, and F in animal feed are regulated [57]. For feed phosphates, the limits are as follows [in mg/kg]: Hg: 0.1; F: 2000; Cd: 10 [57].

IFPs are not pure compounds. Calcium phosphates typically contain three types of molecules: MCP, DCP, and TCP, and the chemical form depends on the relative proportion of each. According to [58], a mono-constituent substance contains a single constituent at a concentration of at least 80%, with up to 20% impurities. The substance is named according to its main constituent. For example, in the REACH registration, the MCP molecule (Ca(H_2_PO_4_)_2_·H_2_O) constitutes over 80%, whereas the DCP molecule (CaHPO_4_) is considered an impurity with a maximum concentration of 20%.

In vivo tests are essential for accurately determining phosphate digestibility or availability, but they cannot be conducted routinely. Therefore, rapid in vitro P solubility analyses are recommended to validate phosphate quality [59]. The most suitable method for rapid estimation of P availability is the 2% citric acid solubility test, which is commonly used. Solubility in alkaline ammonium citrate, however, provides information on the chemical character of the product and the presence of TCP molecules. Phosphates with >90% solubility in both tests are considered to have high nutritional quality [54].

Phosphorus water solubility (Pws) provides insight into the molecular composition of phosphates, as it is positively correlated with MCP content in calcium phosphates and, consequently, with digestibility [60]. Higher solubility corresponds to higher digestibility [54].

The relative P bioavailability [29,61] of five IFP sources was tested in growing pigs. The IFP sources included DCP, MCP containing 50% MCP (MCP50), MCP containing 70% MCP (MCP70), MCP containing 100% MCP (MCP100), and monosodium phosphate (MSP). Results indicated that P in MSP and MCP100 is more bioavailable than P in DCP, whereas no significant differences were observed among the different MCP sources.

A detailed characterization of feed phosphates is presented in Table 7.

## 4. Preparation of MCP from Hydroxyapatite Ashes Obtained from the Thermal Processing of MBM

Drying is a crucial phase that significantly affects the quality of inorganic feed phosphates (IFPs). Excessively high drying temperatures can cause monocalcium phosphate (MCP) to lose its water of crystallization at 135–170 °C, while calcium metaphosphates form at approximately 270 °C. Dicalcium phosphate (DCP) begins to lose its water of crystallization at 85 °C, initially slowly, and then more rapidly at 174 °C, with complete water loss occurring at 213 °C. At 420–430 °C, CaHPO_4_ loses its constitutional water, forming calcium pyrophosphate. Conversely, drying at too low temperatures may cause MCP to decompose into DCP and free acid. In both cases, the resulting product becomes viscous, complicating sieving [62].

Studies on MCP production from hydroxyapatite (HA) obtained after thermal treatment of meat-bone meal (MBM) were carried out using a developed low-temperature method. MCP is produced according to reaction (1):

Ca_5_(PO_4_)_3_(OH) + 7 H_3_PO_4_ + 4 H_2_O = 5 Ca(H_2_PO_4_)_2_∙H_2_O (1)

Based on this reaction, tests were conducted to produce monocalcium phosphate (MCP). The process involved mixing hydroxyapatite ash (HA) with phosphoric acid (H_3_PO_4_) in a stoichiometric ratio and grinding the mixture in a mortar according to three variants:

- Variant A: HA was ground with H_3_PO_4_, without the addition of recycled MCP.
- Variant B: HA was ground with H_3_PO_4_, followed by the addition of recycled MCP, and the mixture was ground again.
- Variant C: HA was mixed with recycled MCP and then ground with H_3_PO_4_.

The obtained samples were subsequently dried for 1 h at 105 °C. Total phosphorus and available phosphorus contents were determined spectrophotometrically. Calcium content and X-ray phase composition were also analyzed (Table 8). X-ray diffraction analysis showed that, in all cases, the primary crystalline phase is monocalcium phosphate (Ca(H_2_PO_4_)_2_·H_2_O), while hydroxyapatite (Ca_10_(PO_4_)_6_(OH)_2_) is present as a minor phase (Figures S1–S3).

Next, tests were conducted by mixing hydroxyapatite (HA), obtained by calcining MBM at 950 °C for 3 h, with a stoichiometric amount of 75% phosphoric acid (H_3_PO_4_) and grinding the mixture in a mortar. The resulting material was very difficult to grind, as it hardened rapidly. The product, dried at 105 °C for 1 h, was lumpy and very hard.

**Figure 11:**
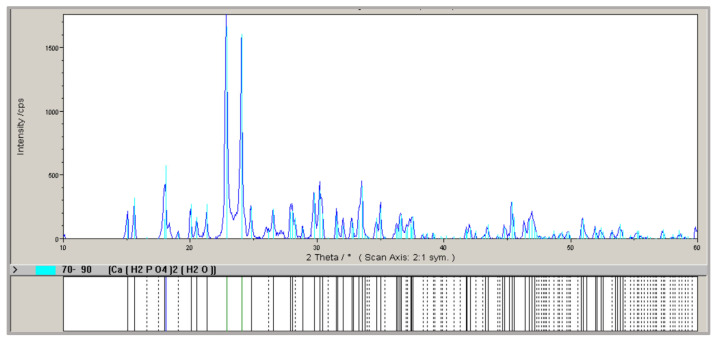
X-ray diagram of the monocalcium phosphate obtained in test 0(1).

In test (1), 10.02 g of HA and 16.83 g of H_3_PO_4_ were mixed, yielding 22.08 g of product after drying. In test (2), 10.02 g of HA and 16.79 g of H_3_PO_4_ were mixed, producing 23.60 g of MCP. X-ray diffraction analysis of the MCP obtained in test (1) (Figure 11) identified monocalcium phosphate (Ca(H_2_PO_4_)_2_·H_2_O) as the crystalline phase.

### Preparation of MCP with Recycling of the Final Product

Hydroxyapatite (HA), obtained by calcining MBM at 950 °C for 3 h, was mixed with a stoichiometric amount of 75% phosphoric acid (H_3_PO_4_) and various amounts of recycled MCP, and then ground in a mortar. Two variants for adding the recycled product were tested:

- Variant A: HA was mixed with recycled MCP, followed by the addition of H_3_PO_4_.
- Variant B: HA was mixed with H_3_PO_4_, followed by the addition of recycled MCP.

In Variant A, adding the acid to the HA–MCP mixture made grinding easier, and the resulting masses did not stick together. The products were more free-flowing and easier to handle. In Variant B, grinding the HA with acid initially was difficult, producing very sticky masses. However, after adding the recycled MCP, grinding became easier, resulting in a loose, fine product. Variant A proved to be the most advantageous, as adding MCP before reaction with H_3_PO_4_ improved the product structure and simplified mechanical processing.

The results presented in Table 9 indicate that the total phosphorus (P) and soluble phosphorus (P in dilute HCl) contents in all samples meet the requirements of the standard [48]. X-ray diffraction analysis of the MCP products confirmed that monocalcium phosphate (MCP) is the primary and exclusive crystalline phase in the samples (Figures S4–S12).

## 5. Flow Sheet of the MCP Industrial Production Process

**Figure 12:**
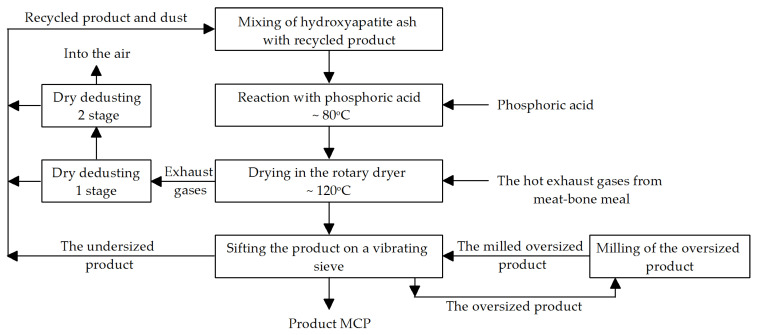
Flow sheet of the monocalcium phosphate (MCP) production process.

The method for preparing MCP from HA involves a series of stages (Figure 12). In the first stage, hydroxyapatite (HA) is mixed with the recycled MCP in a mass ratio of 1:1. The next operation, reaction of this mixture with phosphoric acid, occurs in a mixer, allowing simultaneous mixing and grinding. Afterward, the entire mixture is dried in a rotary dryer at a temperature of up to 120 °C for about 2 h. The dryer is heated with hot exhaust gases from the MBM production process. The drying gases are first dedusted in a cyclone and then in a bag filter, before being released into the atmosphere. Dust from the dust-removal process is returned to the mixing section. The product is screened using a vibrating sieve. Undersized particles are recycled back into the mixing section. Oversized particles (after grinding) are recycled back to the vibrating sieve. The middle fraction constitutes the final product. MCP will be sold in bulk and in 40 kg bags.

### Process Parameters

- Mixing of HA ash with recycled MCP for 20 min at a temperature of up to 50 °C.
- Reaction of the HA/MCP mixture in an Eirich mixer with phosphoric acid: reaction temperature: ~80 °C, continuous dosing of phosphoric acid into the HA/MCP mixture, with continuous stirring, reaction time: ~1 h.
- Drying in a rotary dryer: time: ~2 h (adjustable), consistency of the dried material: coarse paste; operating temperature: up to 150 °C, material temperature <105 °C; co-current operation, 0.2–2.0 rpm; dryer load: average 7 t/h, or ~50 kg/m^2^h.
- Screen operating parameters: temperature around 50 °C.
- Disintegrator operating parameters: temperature 30–40 °C.
- Dry cyclone operating parameters after the dryer: temperature around 100 °C, pressure according to the supplier’s specifications.
- Bag filter operating parameters after the dryer: temperature around 100 °C, pressure according to the supplier’s specifications.

The MCP production unit is strongly connected to the developed MBM unit [36,41], having a production capacity of 30,000 t/y of incinerated MBM. The heat (contained in the hot exhaust gases) and by-product HA from the MBM unit are used in the analyzed MCP installation. From 7500 t/y of HA (obtained after burning 30,000 t/y of MBM), it is possible to obtain 21,700 t/y of MCP. The consumption figures for 1 ton of MCP are: HA—346 kg, 654 kg of 75% H_3_PO_4_ (490 kg of 100% H_3_PO_4_), electricity—26 kWh.

In the conducted study, which was experimental at both laboratory and semi-technical scales, a rotary kiln with an area of 1 m^2^ and a load of 1 kg/m^2^ was used. For comparison, the industrial rotary kiln considered in the analysis has an area of 135 m^2^ and a load of 50 kg/m^2^. The scaling factor for both parameters ranges from 50 to 135, indicating a relatively small difference and suggesting that the transition to industrial scale is technically feasible.

## 6. Preliminary Calculation of MCP Production Costs

Table 10 presents the preliminary production costs of monocalcium phosphate (MCP). The estimated investment expenditures of the MCP unit, having a production capacity of 21,700 tons per year, were $2.5 million. It is assumed that phosphoric acid will be purchased for MCP production at the factual market price in Poland: 75% H_3_PO_4_ at 1231 $/t. The annual demand for 100% acid is 11,940 tons per year. The transport cost for 1 ton of 75% H_3_PO_4_ is assumed to be 20 $. The factual price of MCP, on the Polish market, is 1400 $/t [63].

The integrated process requires only 26 kWh of electricity per ton of MCP containing 22% P (equivalent to 52 kWh per 1 t P_2_O_5_), demonstrating the high energy efficiency of the system. For comparison, the production of DCP (20% P) from phosphoric acid and calcium carbonate consumes 181 kWh and 36 m^3^ of natural gas per 1 t P_2_O_5_, while the production of DFP (18% P) from phosphorites and phosphoric acid requires 206 kWh and 364 m^3^ of natural gas [64].

Additionally, the utilization of waste heat from the combustion of 31,000 t/year of MBM to produce 7500 t of HA ash results in an estimated reduction of approximately 62,000 t of CO_2_ emissions annually [8].

According to market analyses, the global MCP market stood at approximately 3.18 million tons in 2024, with a value of USD 3.85 billion in 2025, and is projected to reach USD 5.94 billion by 2034, growing at a CAGR of 4.9% [63]. The demand for MCP is strongly linked to the expansion of the animal feed industry, driven by the increasing global demand for meat and dairy products, particularly in emerging economies.

MCP prices are influenced by several external factors, including:

- Raw material costs (e.g., phosphate rock, phosphoric acid, energy);
- Global demand and production capacities;
- Geopolitical issues and supply chain disruptions;
- Environmental regulations and sustainability requirements;
- Currency exchange rates and market demand fluctuations [47,63].

While many of these factors are beyond the direct control of producers, they are closely monitored when planning large-scale MCP production. Importantly, in the case of our proposed process, the internal use of approximately 3000 t of MCP per year in Farmutil’s feed plant (with a capacity of ~400,000 t/y) provides an additional buffer against market volatility and ensures that a significant share of production is consumed in-house [47].

Preliminary calculations show that MCP production using the proposed technology is highly profitable. These are results of the close technological and technical integration between the MBM combustion unit and the MCP plant. The advanced industrial symbiosis [47,65] between the MBM and MCP production processes includes, in particular, on-site recycling and reuse of hydroxyapatite—a by-product of MBM combustion—as well as the recovery of free bioenergy in the form of heat from the hot exhaust gases generated during MBM incineration. These solutions are typical of circular economy practices, which are based on cleaner production methods [66].

## 7. Conclusions

The research results present a technology for producing feed-grade MCP from ash obtained through MBM thermal processing, including: characterization of the physico-chemical properties of MBM, determination of parameters for its thermal processing in laboratory- and pilot-scale rotary kilns to obtain high-quality HA ash, and formulation of a conceptual process design for MCP production.

The proposed method for producing monocalcium phosphate (MCP) from hydroxyapatite (HA) ashes, derived from meat-bone meal (MBM) incineration, demonstrates an effective integration of technological and environmental solutions. The process not only recycles HA, a waste product from MBM combustion, but also recovers waste heat from the exhaust gases produced during incineration, providing a significant energy benefit. Energy consumption is minimized, with the process requiring only 26 kWh of electricity per ton of MCP, reflecting the efficiency of the integrated system.

Preliminary economic calculations indicate that the process is highly cost-effective. The production cost of one ton of MCP is $924, while the market price is $1400 per ton, resulting in a profit margin of 34%. For an annual output of 21,700 tons, the total production cost amounts to $20,058,947, with estimated sales revenue of $30,380,000, yielding a profit of $10,321,053.

The proposed MCP production technology aligns with Circular Economy (CE) principles, in which waste products—both HA and energy—are recycled and reused within the production process. This approach supports global sustainable development goals aimed at reducing waste and conserving natural resources. Compared to conventional phosphate manufacturing, the method significantly reduces environmental impact through waste minimization, recovery and reuse of materials and energy, and decreased reliance on primary resources.

The integration of in-process and on-site recycling exemplifies a CE approach, reducing dependence on external raw materials and energy inputs—a key feature of Cleaner Production (CP) principles. By reusing both HA and waste heat, the process substantially lowers the carbon footprint associated with traditional MCP production. The advanced industrial symbiosis between MBM incineration and MCP production offers a promising solution for large-scale phosphate manufacturing, providing economic, environmental, and operational advantages.

## Figures and Tables

**Table 1:** Characteristic of Animal Feed Meals [48].

Features	Physicochemical Requirements of Different Types of Meal					
	MM	MBM	MB	BM	SM	LM
Appearance	Loose, homogeneous, not charred					
Odor	Characteristic, without moldy or musty odors					
Fineness, sieving through a 4 mm square mesh sieve (%)	100					
Moisture, ≤ (%)	10					
Crude fiber content, ≤ (%)	1					
Crude ash content, ≤ (%)	As the producer declares			5.5	As the producer declares	
Total phosphorus content, ≥ (%)	5.5	9	9	Not standardized		
Total Protein Content, ≥ (%)	55.0	40.0	26.5	89.0, 80.0 ^1^	45.0	70.0
Content of Digestible Proteins in total protein, ≥ (%)	87.0	87.0	80.0	90.0	80.0	80.0

^1^ Concerning meals produced using bones. The antioxidant content, expressed as active antioxidants, should be between 100 mg/kg and 400 mg/kg.

**Table 2:** Characteristics of hydroxyapatite ash obtained from meat-bone meal (MBM).

Calcination Temperature of MBM	Weight Loss (%)	P Content (%)	Ca Content (%)	X-Ray Phase Composition
600 °C	70	14.5 ± 0.5 *	33.8 ± 0.4 *	Ca_10_(PO_4_)_6_(OH)_2_, SiO_2_, Ca_3_(PO_4_)_2_, CaCO_3_
950 °C	77	15.0 ± 0.4 *	36.6 ± 0.6 *	Ca_10_(PO_4_)_6_(OH)_2_, SiO_2_, Ca_3_(PO_4_)_2_

* Values ± standard deviation SD.

**Table 3:** Contents of heavy metals and other elements in hydroxyapatite ash HA from incineration of meat and bone meal MBM at 750 °C for 3 h, and in MBM.

Element	MBM		HA After MBM Incineration at 750 °C	
	Content (%)	Uncertainty ± %	Content (%)	Uncertainty ± %
Ca	33.3	6.6	7.95	1.59
K	0.68	0.136	0.452	0.090
Na	1.58	0.32	0.440	0.088
P	4.18	0.84	17.9	3.6
N	8.10	1.60	0.16	0.03
Mg	0.784	0.157	0.199	0.040
	Content (mg/kg)	Uncertainty ± mg/kg	Content (mg/kg)	Uncertainty ± mg/kg
As	<0.010	-	0.84	0.13
Cd	<0.002	-	0.014	0.004
Cu	7.5	1.1	43	6
Fe	3410	680	1010	200
Hg	0.013	0.002	<0.10	-
Pb	1.0	0.1	1.3	0.2
Zn	129	19	189	28
Si	76	11	3410	680
Cl^−^	385	58	146	22
F^−^	117	23	432	86

**Table 4:** Characteristics of hydroxyapatite ashes (HA) produced by calcining mixtures of meat-bone meal (MBM) and recycled hydroxyapatite ash (HA).

Mass Ratio of MBM:HA	P Content (%)	Ca Content (%)
1:4	16.8 ± 0.5 *	36.7 ± 0.8 *
1:5	16.4 ± 0.6 *	36.3 ± 0.7 *
1:6	16.6 ± 0.5 *	35.5 ± 1.0 *
1:7	16.8 ± 0.7 *	36.1 ± 0.7 *
1:8	16.7 ± 0.4 *	36.0 ± 0.6 *
1:9	16.6 ± 0.5 *	37.5 ± 1.0 *
1:10	16.8 ± 0.5 *	35.7 ± 0.5 *

* Values ± standard deviation SD.

**Table 5:** Characteristics of hydroxyapatite ashes (HA) obtained after calcining mixtures of meat-bone meal (MBM) and recycled hydroxyapatite ash (HA) at different parameters.

No	MBM:HA Mass Ratio	Temperature (°C)	Time (min)	P Content (%)	Ca Content (%)
1	1:8.2	879	150	16.67 ± 0.4 *	36.16 ± 0.7 *
2	1:8.2	671	150	16.85 ± 0.5 *	36.05 ± 0.5 *
3	1:2.8	879	150	16.27 ± 0.4 *	36.29 ± 0.8 *
4	1:2.8	671	150	16.30 ± 0.5 *	35.80 ± 0.8 *
5	1:8.2	879	60	16.57 ± 0.5 *	36.59 ± 0.7 *
6	1:8.2	671	60	16.74 ± 0.5 *	35.80 ± 0.6 *
7	1:2.8	879	60	16.72 ± 0.7 *	36.18 ± 0.8 *
8	1:2.8	671	60	16.90 ± 0.4 *	37.05 ± 1.0 *
9	1:1	775	105	16.36 ± 0.5 *	34.99 ± 0.8 *
10	1:10	775	105	16.80 ± 0.5 *	35.24 ± 1.0 *
11	1:5.5	600	105	16.69 ± 0.6 *	35.70 ± 1.1 *
12	1:5.5	950	105	17.27 ± 0.4 *	35.93 ± 0.9 *
13	1:5.5	775	30	17.02 ± 0.5 *	35.69 ± 0.8 *
14	1:5.5	775	180	16.93 ± 0.6 *	35.49 ± 0.7 *
15	1:5.5	775	105	16.58 ± 0.7 *	36.11 ± 1.1 *
16	1:5.5	775	105	16.66 ± 0.5 *	35.82 ± 0.9 *
17	1:5.5	775	105	16.86 ± 0.5 *	35.30 ± 1.0 *
18	1:5.5	775	105	16.74 ± 0.4 *	36.43 ± 1.0 *
19	1:5.5	775	105	17.07 ± 0.5 *	35.67 ± 0.6 *
20	1:5.5	775	105	17.03 ± 0.6 *	35.77 ± 0.8 *

* Values ± standard deviation SD.

**Table 6:** Characteristics of hydroxyapatite ash (HA) product of calcining meat-bone meal (MBM) in rotary kiln.

Test	Temperature (°C)	Mass Ratio of MBM:HA	P Content (%)	Ca Content (%)	X-Ray Phase Composition
1	600	1:1	16.11 ± 0.4 *	36.49 ± 0.9 *	Ca_10_(PO_4_)_6_(OH)_2_
2	600	1:2	14.98 ± 0.5 *	37.29 ± 0.7 *	Ca_10_(PO_4_)_6_(OH)_2_
3	600	1:3	16.70 ± 0.6 *	36.95 ± 0.8 *	Ca_10_(PO_4_)_6_(OH)_2_
4	800	1:1	17.42 ± 0.7 *	36.97 ± 1.0 *	Ca_10_(PO_4_)_6_(OH)_2_
5	800	1:2	16.95 ± 0.5 *	36.71 ± 0.7 *	Ca_10_(PO_4_)_6_(OH)_2_
6	800	1:3	16.49 ± 0.6 *	37.11 ± 0.6 *	Ca_10_(PO_4_)_6_(OH)_2_

* Values ± standard deviation SD.

**Table 7:** Detailed characterization of feed phosphates [48].

Features	Feed Phosphate Type					
	Monocalcium	Dicalcium	Tricalcium	Calcium-Sodium	Sodium Calcium Magnesium	Ammonium
Appearance	Loose or granulated					
Odor and color	specific					
Fineness: loose phosphates—residue on a sieve with a mesh of 0.3 mm, ≤ (%)	10					
Granulated and loose phosphates, sifting throughout a sieve mesh of 3 mm, (%)	100					
Phosphorus content, ≤ (%)	22	16	18	16	17	25
Phosphorus content, soluble in 0.4% HCl solution, ≤ (%)	20.0	14.5	16.0	14.5	15.3	22.5
Calcium content, (%)	15–20	21–30	31–35	12–26	5–10	
Sodium content, (%)				6–8	11–14	
Magnesium content, ≤ (%)					3	
Nitrogen content, %						11–12
Chlorides as NaCl, ≤ (%)		1	1			
Fluorine content, ≤ (%)	0.2					
Lead content, ≤ (%)	0.0030					
Cadmium content, ≤ (%)	0.0010					
Mercury content, ≤ (%)	0.00010					
Arsenic content, ≤ (%)	0.0010					

**Table 8:** Characteristics of the produced monocalcium phosphate samples.

Variant	P (%)			Ca Content (%)	X-Ray Phase Composition
	Total Content	Solubility in 0.4% HCl	Solubility in 2% Citric Acid		
A	18.5 ± 0.4 *	100 ± 0.8 *	100 ± 0.7 *	16.0 ± 0.5 *	Ca(H_2_PO_4_)_2_ · H_2_O, Ca_10_(PO_4_)_6_(OH)_2_
B	21.9 ± 0.5 *	100 ± 0.8 *	100 ± 0.9 *	16.5 ± 0.4 *	Ca(H_2_PO_4_)_2_ · H_2_O, Ca_10_(PO_4_)_6_(OH)_2_
C	22.5 ± 0.5 *	100 ± 0.6 *	100 ± 0.8 *	16.5 ± 0.4 *	Ca(H_2_PO_4_)_2_ · H_2_O, Ca_10_(PO_4_)_6_(OH)_2_

* Values ± standard deviation SD.

**Table 9:** Results of tests on two variants of adding the recycled product: A. HA mixed with recycled MCP, followed by the addition of phosphoric acid; B. HA mixed with phosphoric acid, followed by the addition of recycled MCP.

Recycled MCP:HA Mass Ratio	Test	P Content (%)			Ca Content (%)	X-Ray Phase Composition
		Total	Soluble in 0.4% HCl	Soluble in 2% Citric Acid		
0	0(1)	23.5 ± 0.4 *	23.3 ± 0.8 *	23.5 ± 0.6 *	15.1 ± 0.4 *	Ca(H_2_PO_4_)_2_·H_2_O
	0(2)	23.5 ± 0.5 *	23.4 ± 0.7 *	23.5 ± 0.5 *	14.9 ± 0.3 *	Ca(H_2_PO_4_)_2_·H_2_O
0.5	0.5A	24.0 ± 0.5 *	22.0 ± 0.4 *	23.2 ± 0.5 *	16.4 ± 0.4 *	Ca(H_2_PO_4_)_2_·H_2_O
	0.5B	24.3 ± 0.7 *	22.6 ± 0.6 *	23.6 ± 0.5 *	15.9 ± 0.7 *	Ca(H_2_PO_4_)_2_·H_2_O
1	1A(1)	24.0 ± 0.4 *	22.8 ± 0.5 *	23.3 ± 0.7 *	14.4 ± 0.4 *	Ca(H_2_PO_4_)_2_·H_2_O
	1A(2)	23.7 ± 0.7 *	22.8 ± 0.6 *	24.0 ± 0.5 *	15.4 ± 0.6 *	Ca(H_2_PO_4_)_2_·H_2_O
	1B(1)	23.9 ± 0.7 *	21.8 ± 0.6 *	23.9 ± 0.5 *	15.1 ± 0.6 *	Ca(H_2_PO_4_)_2_·H_2_O
	1B(2)	23.2 ± 0.4 *	22.4 ± 0.4 *	23.6 ± 0.3 *	14.8 ± 0.3 *	Ca(H_2_PO_4_)_2_·H_2_O
	1A(H)	23.0 ± 0.5 *	21.9 ± 0.4 *	22.6 ± 0.3 *	14.8 ± 0.5 *	Ca(H_2_PO_4_)_2_·H_2_O
	1B(H)	22.9 ± 0.6 *	22.5 ± 0.7 *	23.1 ± 0.5 *	16.0 ± 0.6 *	Ca(H_2_PO_4_)_2_·H_2_O
2	2A	23.7 ± 0.7 *	22.3 ± 0.5 *	23.2 ± 0.4 *	15.7 ± 0.5 *	Ca(H_2_PO_4_)_2_·H_2_O
	2B	23.9 ± 0.6 *	22.0 ± 0.6 *	23.3 ± 0.5 *	16.1 ± 0.7 *	Ca(H_2_PO_4_)_2_·H_2_O
3	3A	24.4 ± 0.3 *	23.0 ± 0.5 *	23.8 ± 0.7 *	15.5 ± 0.5 *	Ca(H_2_PO_4_)_2_·H_2_O
	3B	23.6 ± 0.6 *	22.7 ± 0.5 *	24.1 ± 0.5 *	15.0 ± 0.4 *	Ca(H_2_PO_4_)_2_·H_2_O
4	4A	24.3 ± 0.5 *	22.6 ± 0.6 *	24.0 ± 0.4 *	15.1 ± 0.6 *	Ca(H_2_PO_4_)_2_·H_2_O
	4B	24.2± 0.4 *	22.4 ± 0.8 *	24.6 ± 0.5 *	14.9 ± 0.7 *	Ca(H_2_PO_4_)_2_·H_2_O
5	5A	24.5 ± 0.7 *	22.2 ± 0.5 *	24.5 ± 0.7 *	14.8 ± 0.5 *	Ca(H_2_PO_4_)_2_·H_2_O
	5B	24.3 ± 0.5 *	22.5 ± 0.4 *	23.7 ± 0.6 *	15.0 ± 0.7 *	Ca(H_2_PO_4_)_2_·H_2_O

* Values ± standard deviation SD.

**Table 10:** Preliminary MCP production costs for monocalcium phosphate.

No	Calculation Position	Unit	Consumption Figure	Price per Unit ($)	Cost per	
					1 t ($)	Year ($)
1	Direct materials				825	17,902,500
	75% H_3_PO_4_	kg/t	654			
	100% H_3_PO_4_	kg/t	490	1642	805	17,468,500
2	Purchase costs					
	75% H_3_PO_4_	kg/t	654	31	20	434,000
3	Total material costs (items 1–2)				825	
4	Own semi-finished products				-	
	Hydroxyapatite ash	kg/t	346	-	-	
5	Process energy				4.2	
	- electricity	kWh/t	26	0.16	4.2	
6	Direct salaries				30.14	
7	Total direct costs (items 3–6)				829	
8	Chemical analyses costs				3	65,100
9	Environmental Use Fees				0.5	10,850
10	Variable line costs (items 7–9)				833	18,069,590
11	Maintaining Machinery and Equipment				8.65	189,000
	- repairs and maintenance				0.91	21,000
	- amortization 8%				7.74	168,000
	- auxiliary materials				-	
12	Total Production Cost at Plant (items 10–11)				841	18,258,590
13	Labor resources maintenance, including:				0.5	10,850
	- occupational health & safety costs				0.5	10,850
14	Plant’s fixed costs				4.35	100,000
15	General production process management				4.7	102,000
	- technical supervisors’ salaries (1700 $/person × 4 employees/month + 25% overhead)				4.7	102,000
16	Total Net Production Cost at the Plant (items 12–15)				851	18,471,440
17	Collected for further processing (item 16)				851	18,471,440
18	Cost of product without packaging (item 17)				851	18,471,440
19	Packaging				-	-
	- paper bags (25 kg)	pieces/t	40	0.50	20	434,000
20	Main product manufacturing cost (items 18–19)				871	18,905,440
21	Total administrative costs	%		3	26	567,021
22	Factory manufacturing cost (items 20–21)				897	19,472,461
23	Cost of sales				27	586,486
22	Total production costs (items 22–23)				924	20,058,947
25	MCP sales revenue			1400	1400	30,380,000
26	Profit				$	10,321,053
	Profit margin				%	34

## Data Availability

The original contributions presented in this study are included in the article/Supplementary Material. Further inquiries can be directed to the corresponding author.

## Supplementary Materials

The following supporting information can be downloaded at: https://www.mdpi.com/article/10.3390/ma18204653/s1, Figure S1: X-ray diagram of monocalcium phosphate obtained according to Variant A (designated in Table 8); Figure S2: X-ray diagram of monocalcium phosphate obtained according to Variant B (designated in Table 8); Figure S3: X-ray diagram of monocalcium phosphate obtained according to Variant C (designated in Table 8); Figure S4: X-ray diagram of monocalcium phosphate obtained according to test 0(1) (designated in Table 9); Figure S5: X-ray diagram of monocalcium phosphate obtained according to test 0.5A (designated in Table 9); Figure S6: X-ray diagram of monocalcium phosphate obtained according to test 1A(2) (designated in Table 9); Figure S7: X-ray diagram of monocalcium phosphate obtained according to test 1B(2) (designated in Table 9); Figure S8: X-ray diagram of monocalcium phosphate obtained according to test 1A(H) (designated in Table 9); Figure S9: X-ray diagram of monocalcium phosphate obtained according to test 2A (designated in Table 9); Figure S10: X-ray diagram of monocalcium phosphate obtained according to test 3A (designated in Table 9); Figure S11: X-ray diagram of monocalcium phosphate obtained according to test 4A (designated in Table 9); Figure S12: X-ray diagram of monocalcium phosphate obtained according to test 5A (designated in Table 9).

## Abbreviations

The following abbreviations are used in this manuscript:

MBM	Meat-bone meal
MM	Meat meal
MCP	Monocalcium phosphate
DCP	Dicalcium phosphate
TCP	Tricalcium phosphate
DFP	Defluorinated calcium phosphate
HA	Hydroxyapatite
IS	Industrial symbiosis
CE	Circular economy
CP	Cleaner production
SD	Sustainable development
IFP	Inorganic feed phosphates
BM	Blood meal
MB	Bone meal
SM	Skin Meal
LM	Liquex meal
