# Supplementary material for: Production of Food-Grade Monocalcium Phosphate from Meat-Bone Meal
Source: Materials (Basel). 2025 Oct 10;18(20):4653. doi: 10.3390/ma18204653 (PMC12565605; doi:10.3390/ma18204653)
Supplement: Supplementary file 1 [file materials-18-04653-s001.zip › materials-3884926-supplementary.pdf]

**Production of Food-Grade Monocalcium Phosphate from Meat-Bone Meal**

Zygmunt Kowalski [1,*], Agnieszka Wilkosz-Jężyk [2] and Agnieszka Makara [2]

[1]Mineral and Energy Economy Research Institute of the Polish Academy of Sciences, Wybickiego 7A, 31-261 Kraków, Poland; zkow@meeri.pl

[2]Cracow University of Technology, Faculty of Chemical Engineering and Technology, Warszawska 24, 31-155 Kraków, Poland; a.wilkosz.jezyk@gmail.com (A.W-J.); agnieszka.makara@pk.edu.pl (A.M.)

*Correspondence: zkow@meeri.pl; Tel.: +48-12-617-16-33

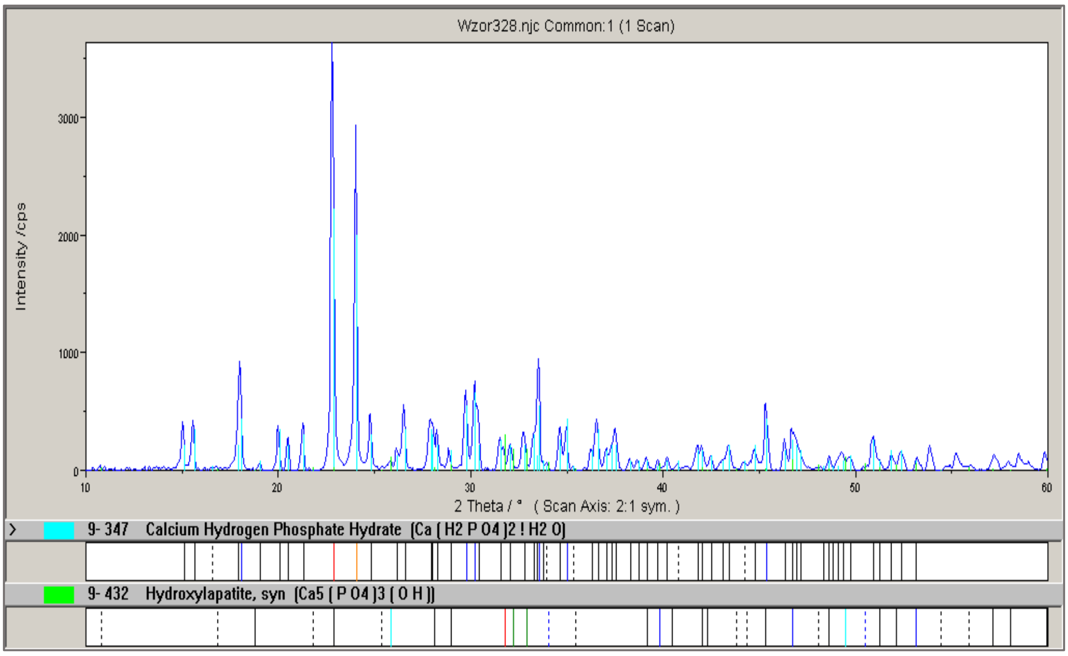


Fig.S1 X-ray diagram of monocalcium phosphate obtained according to Variant A (designated in Table 8).

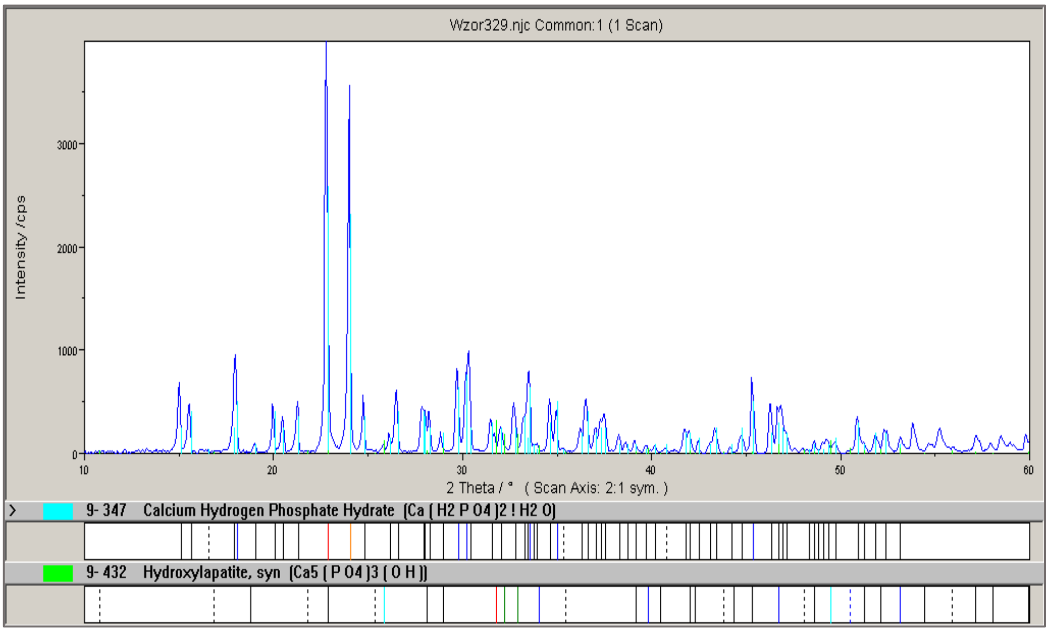


Fig. S2 X-ray diagram of monocalcium phosphate obtained according to Variant B (designated in Table 8).

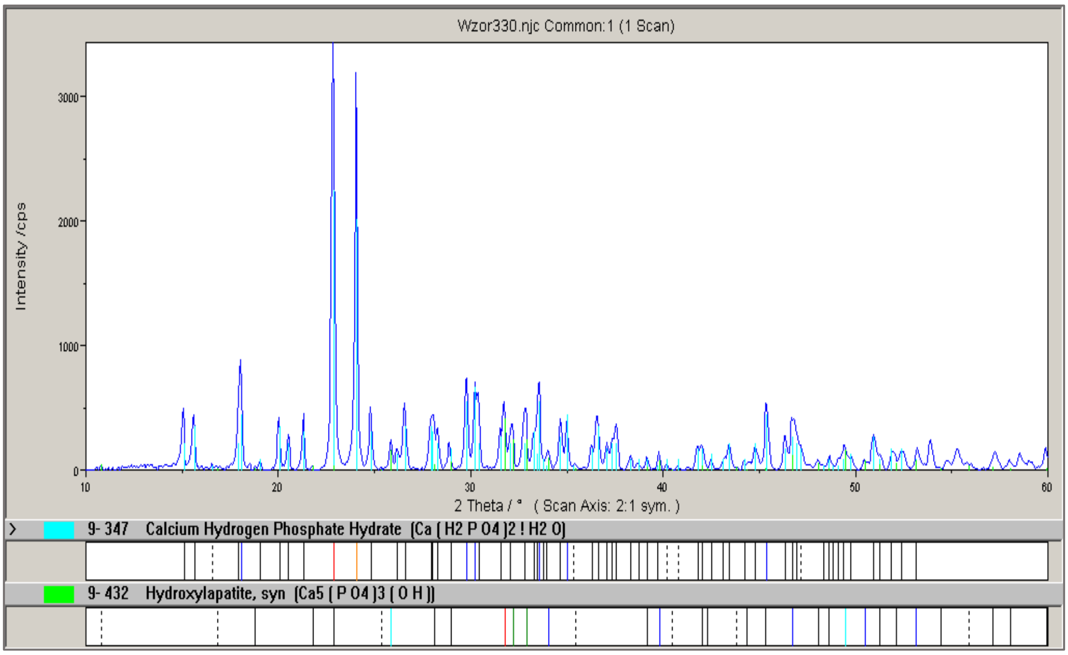


Fig. S3 X-ray diagram of monocalcium phosphate obtained according to Variant C (designated in Table 8).

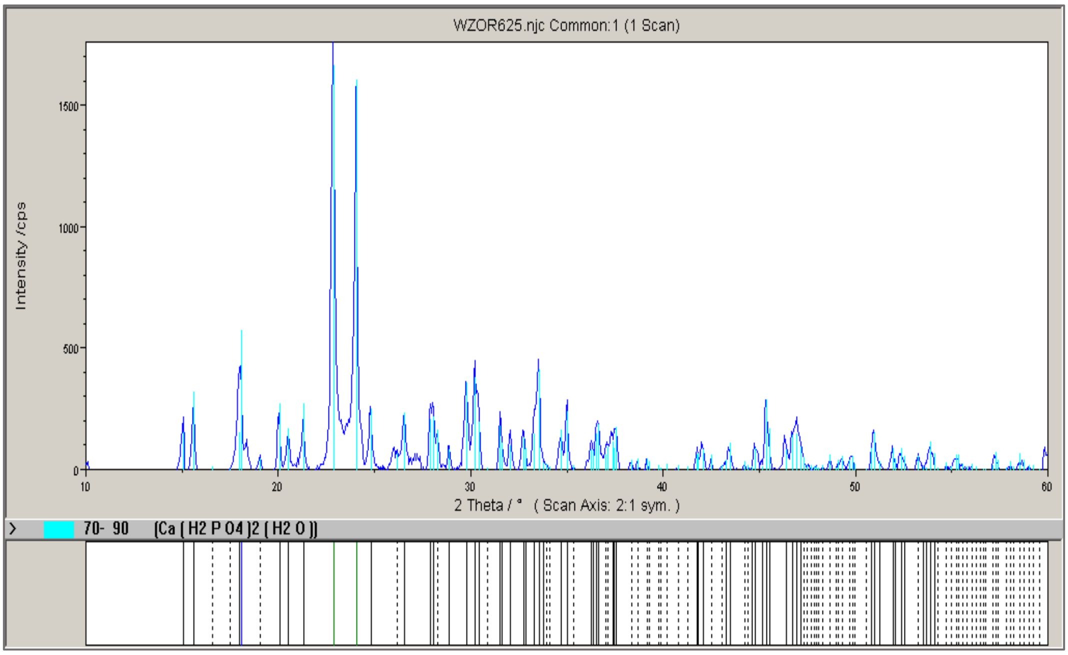


Fig. S4 X-ray diagram of monocalcium phosphate obtained according to test 0(1) (designated in Table 9).

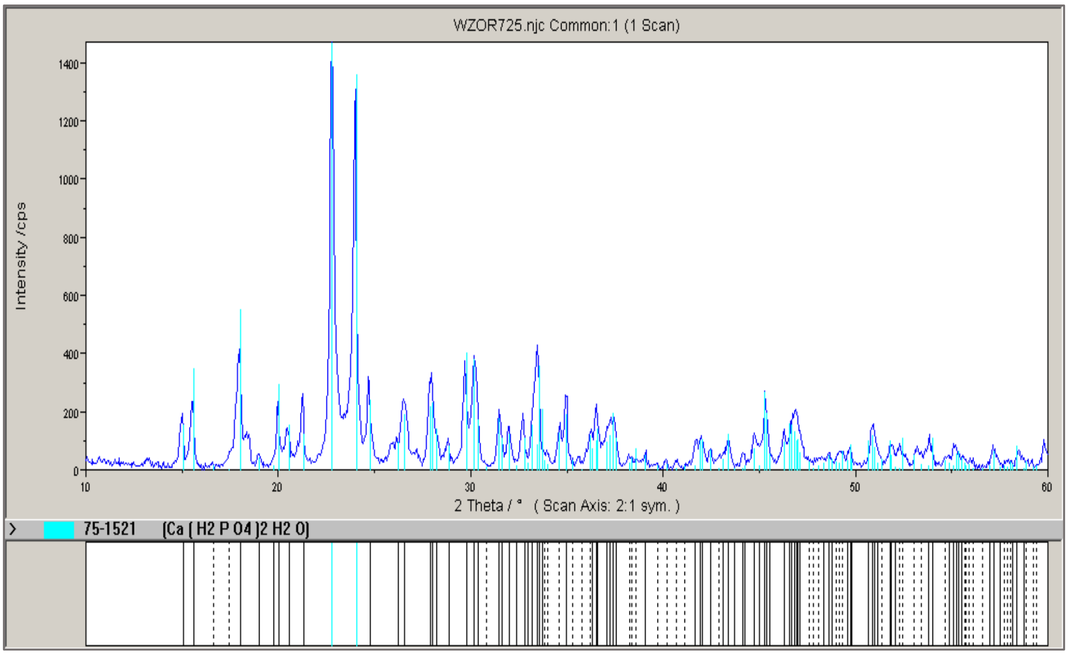


Fig. S5 X-ray diagram of monocalcium phosphate obtained according to test 0.5A (designated in Table 9).

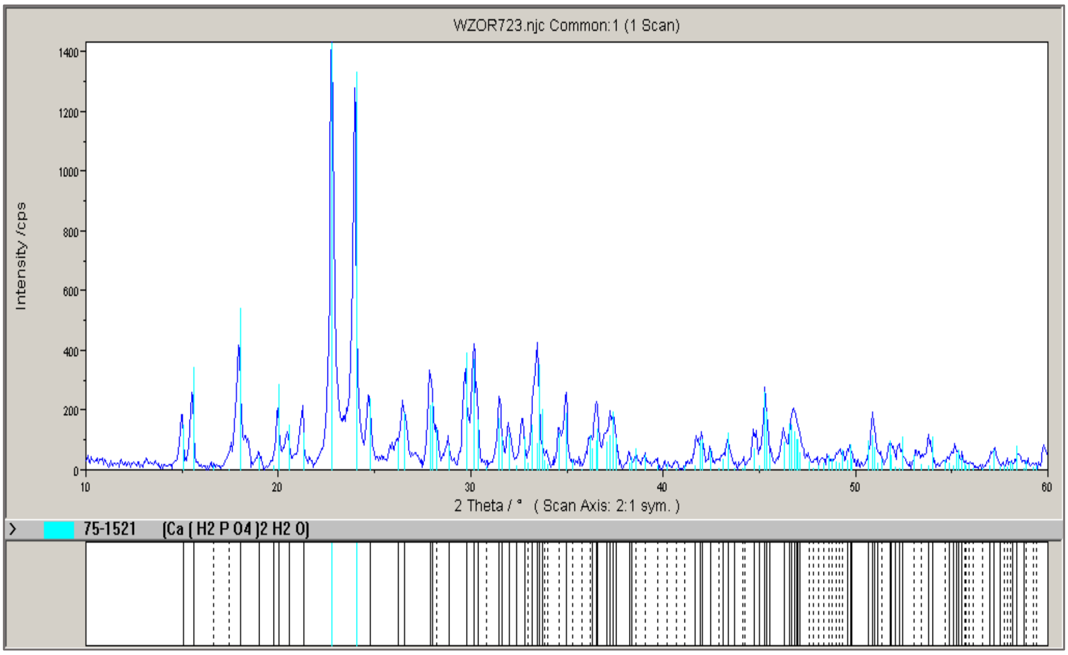


Fig. S6 X-ray diagram of monocalcium phosphate obtained according to test 1A(2) (designated in Table 9).

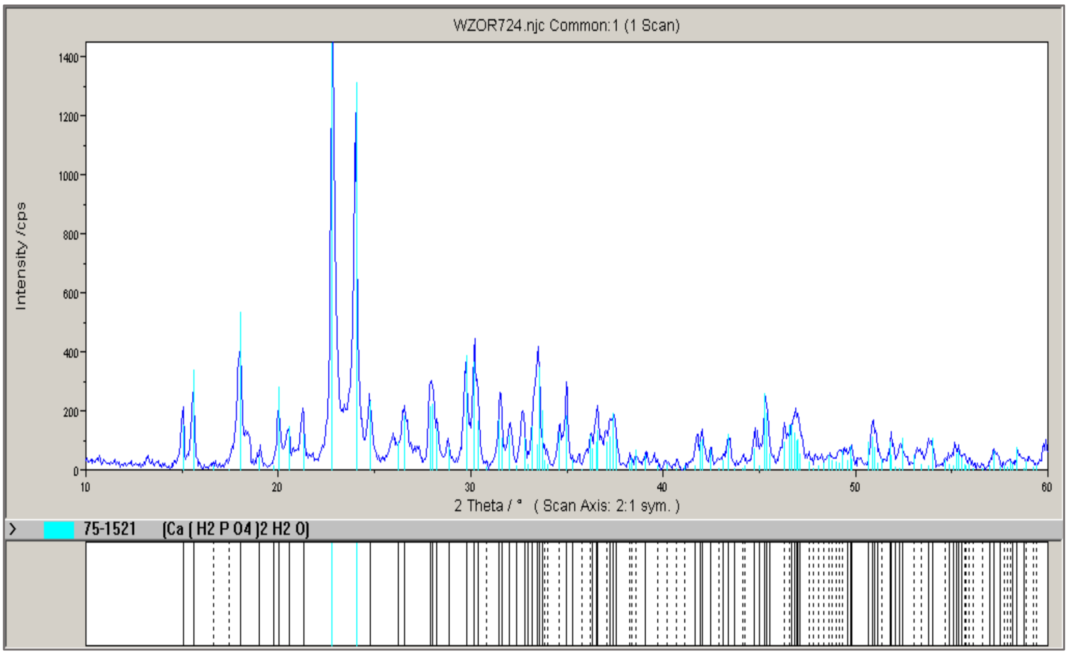


Fig. S7 X-ray diagram of monocalcium phosphate obtained according to test 1B(2) (designated in Table 9).

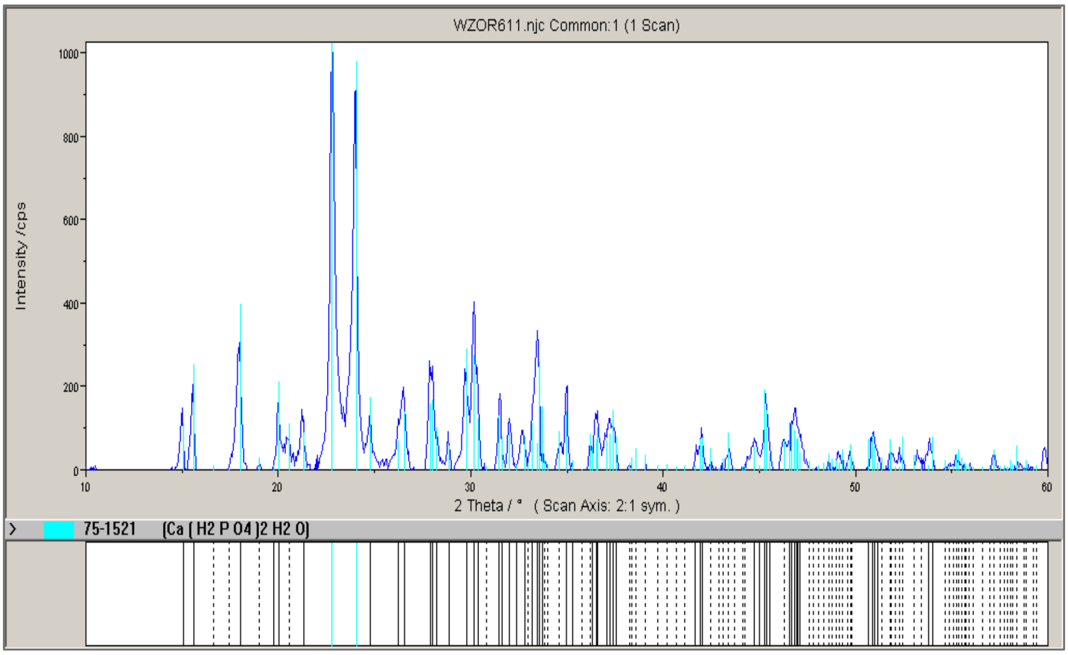


Fig. S8 X-ray diagram of monocalcium phosphate obtained according to test 1A(H) (designated in Table 9).

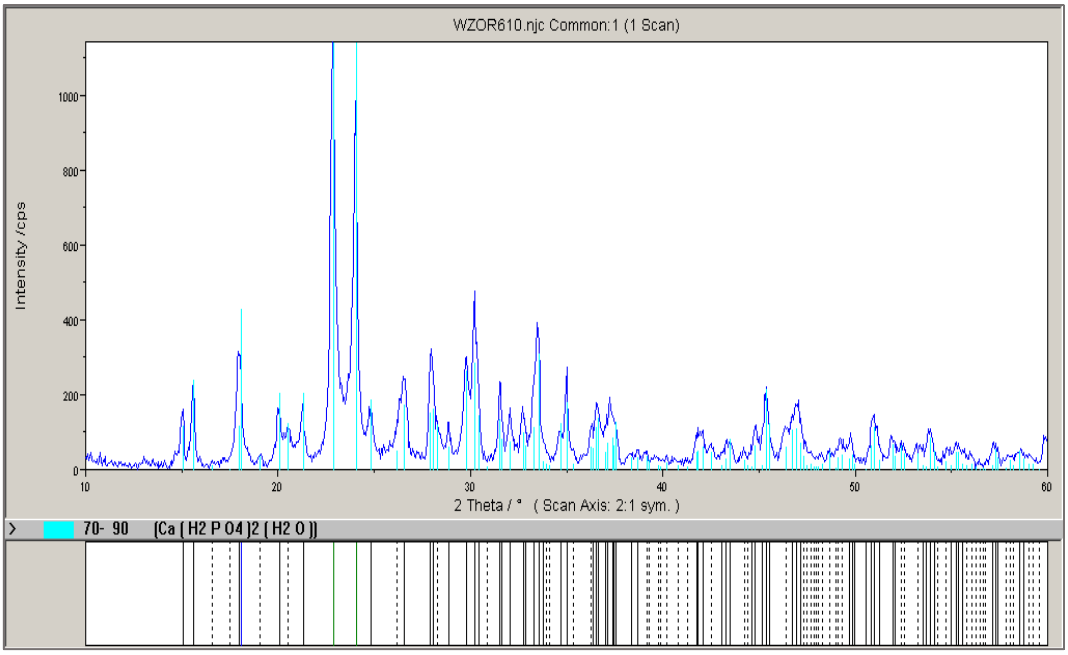


Fig. S9 X-ray diagram of monocalcium phosphate obtained according to test 2A (designated in Table 9).

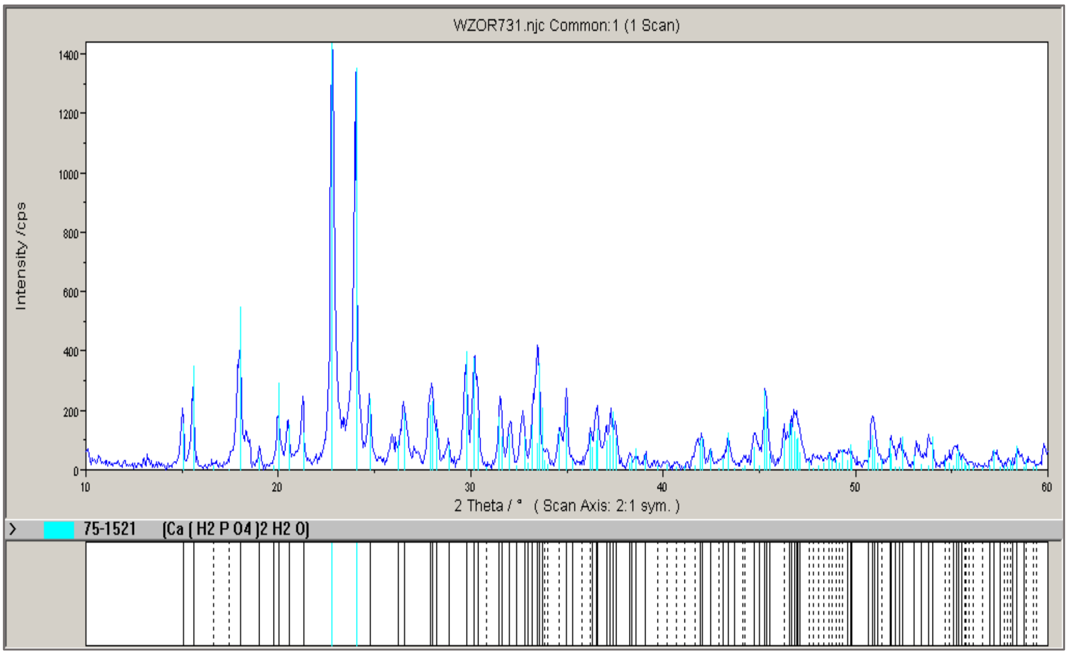


Fig. S10 X-ray diagram of monocalcium phosphate obtained according to test 3A (designated in Table 9).

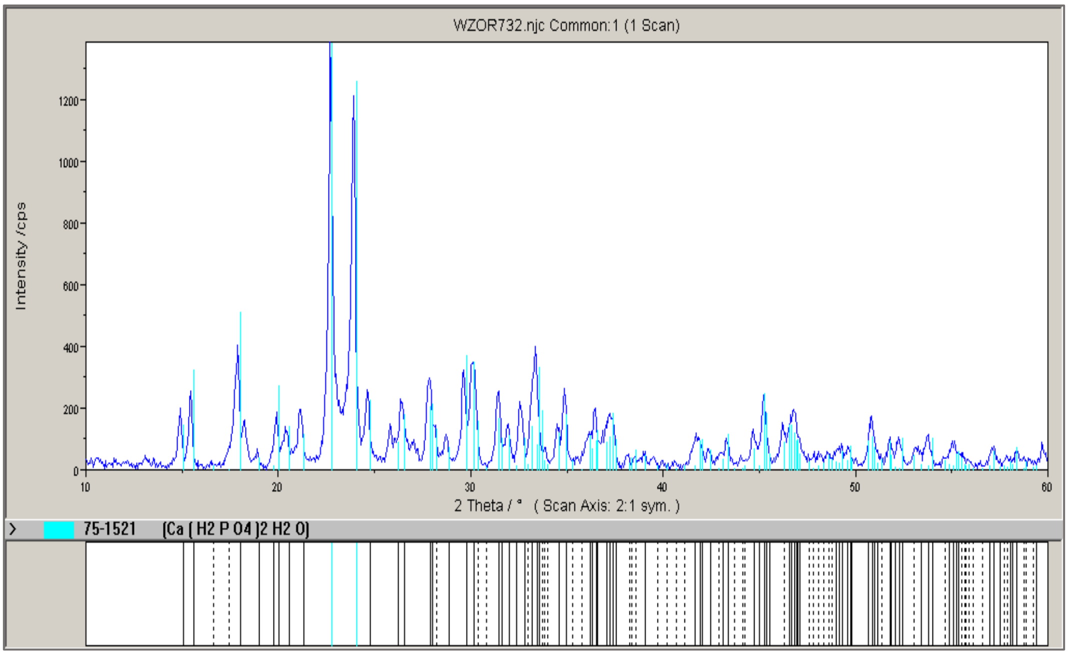


Fig. S11 X-ray diagram of monocalcium phosphate obtained according to test 4A (designated in Table 9).

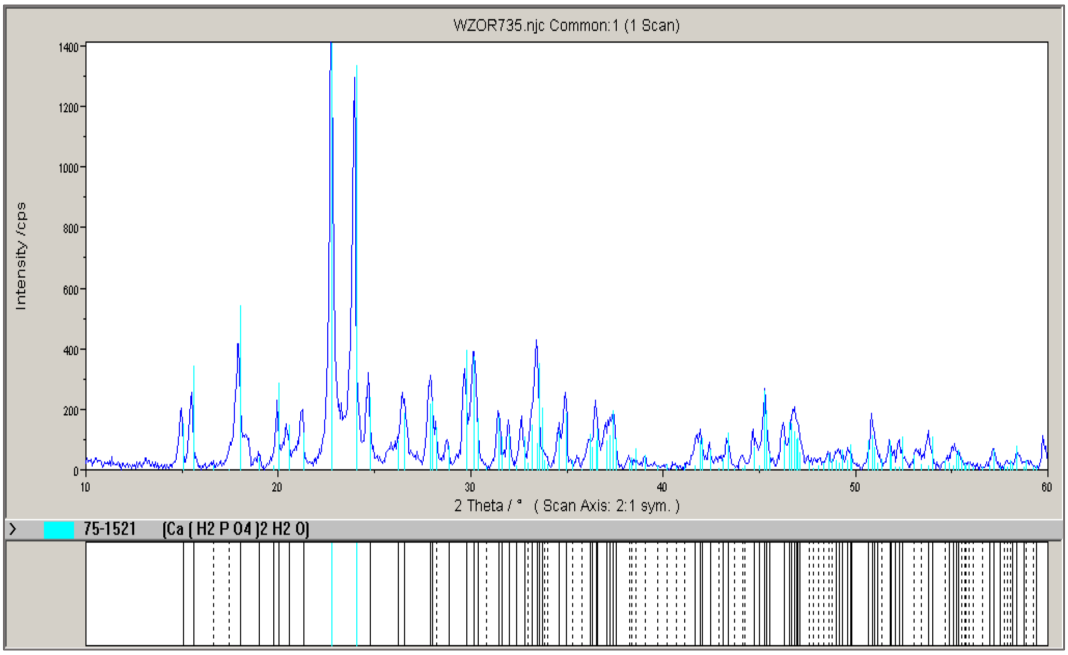


Fig. S12 X-ray diagram of monocalcium phosphate obtained according to test 5A (designated in Table 9).
